# The racer's gaze: Visual strategy in high-speed sports expertise

**DOI:** 10.1167/jov.25.8.16

**Published:** 2025-07-21

**Authors:** Otto Lappi, Jami Pekkanen, Aleksandra Krajnc, Lucas Iacono, Adrian Remonda, Eduardo Veas

**Affiliations:** 1 Cognitive Science, Department of Digital Humanities, University of Helsinki, Helsinki, Finland; 2 Know Center Research GmbH, Graz, Austria; 3 AVL List GmbH, Graz, Austria; 4 Interactive Systems and Data Science, Graz University of Technology, Graz, Austria

**Keywords:** visuomotor coordination, perceptual–cognitive expertise, gaze strategies, naturalistic tasks, sports, methodology

## Abstract

Eye movements shape all visual input to the brain, making their understanding essential for studying perception and visual guidance in dynamic environments. Research on expert performance indicates that gaze coordination is a key feature of expertise in, for example, sports. Mobile eye tracking provides the opportunity to investigate gaze strategies supporting the skilled actions of an athlete and can deliver insight into the underlying perceptual–cognitive processes. We systematically observed the visual strategy of an expert racing driver performing a domain-representative task. Synchronized gaze, telemetry, and localization data from a high-grade simulator were analyzed to address four classes of research questions: oculomotor, scene analysis, timing, and point of vantage. The results (a) replicate the seminal tangent point orientation (pre–turn-in saccades), (b) describe both the oculomotor signature and timing signature of the steering with the head strategy, (c) identify a novel saccade strategy (pre–full-throttle saccades), and (d) reveal a previously unstudied spatial regularity in the serial organization of behavior: a tight localization of the points of vantage where the pre–turn-in saccades and pre–full-throttle saccades are made. The gaze strategies are not tied to specifics of the task and may be relevant for understanding expert performance in other fields with similar visuomotor and cognitive demands. The method of cross-examining an integrated dataset by multiple parametrizations itself complements traditional research designs with predefined task constraints and restrictions. We are not aware of any study that has simultaneously addressed all four kinds of research questions simultaneously.

## Introduction

Eye movements modulate all visual input to the brain. Understanding them is therefore essential for understanding all aspects of visual brain function, such as perception, attention, memory, and dynamic real-world decision making. In dynamic tasks, active gaze partially determines when, from where, and for how long visual information gets sampled. For neuroscientists who want to venture outside of the lab, mobile eye tracking offers means to investigate how active gaze is deployed “in the wild.”^1^

Studies on experts have shown that specific gaze strategies (often different from those of novices) support their exceptional perceptual–cognitive capacities. Eye tracking can be combined with performance analysis to gain insight into these highly dynamic behaviors, and into the visuomotor and cognitive processes behind them. An expert engaging in representative task performance in a controlled setting therefore offers an attractive model system to understand many aspects of natural tasks.

Sports, especially, are in many ways an ideal test bed where we can set up repeatable, highly practiced, and ecologically valid tasks (Williams & Ericsson, 2005; Yarrow, Brown, & Krakauer, 2009; Walsh, 2014; Mangalam, Yarossi, Furmanek, Krakauer, & Tunik, 2023), and investigating the gaze strategies of skilled athletes has indeed proven to be a useful tool to probe the underlying perceptual–cognitive processes (Land & Tatler, 2009; Vickers, 2016; Kredel, Vater, Klostermann, & Hossner, 2017; Kredel, Hernandez, Hossner, & Zahno, 2023). In high-speed sports—where the athlete achieves speeds beyond those limited by the force or power they are capable of producing using muscle force alone, pushing human sensorimotor, physiological, and perceptual–cognitive processes to the limit—accurate anticipatory visual guidance becomes critical, and time constraints leave no room for any inessential gaze behaviors. Here, we combined synchronized localization, telemetry, and gaze data collected from an expert racing driver in a high-grade, industry gold-standard racing simulator, a far richer and more demanding task than most oculomotor experiments in the laboratory.

Previous research on expert gaze strategies in the high-speed sports domain (Vickers, 2006; Decroix et al., 2017), including expert race driving (Land & Tatler, 2001; van Leeuwen, de Groot, Happee, & de Winter, 2017; Lima, Haar, Di Grassi, & Faisal, 2020, Nishizono, Saijo, & Kashino, 2023), has posed and begun to answer many outstanding questions: *What visual cues in the scene do experts pick up and use? How do specific eye movements glean information about them? How is the timing of gaze (eye and head) and locomotor control (limbs) coordinated?*

These questions are different ways of looking at naturalistic gaze data, highlighting different phenomena in the underlying behavior and leading us to pose different questions to the data. The main novelty of our study is that we addressed a fourth (rarely posed) question: *Where in space are gaze actions performed, relative to locomotor actions and outcomes?* For parametrizing visual strategies in high-speed sports, this represents a complementary approach to the other three questions—one that only makes sense in mobile contexts and, perhaps because of that, is much more rarely posed or recognized as a distinct research problem. Here, we introduce keypoint-based point of vantage analysis and illustrate its use and usefulness in analyzing the active gaze strategy of an expert. We then discuss what makes it conceptually and methodologically distinct and the novel points of view and phenomena it can reveal in other high-speed sports and locomotor contexts.

High-speed visual guidance of self-motion in the three-dimensional (3D) scene is a fundamental problem for the visual system. As such, task performance is likely supported by highly efficient, evolved, visuomotor and perceptual–cognitive mechanisms for encoding 3D scene information, transforming it into a coherent perception and memory representations, and deploying these in executing well-coordinated motor actions (Land & Furneaux, 1997; Land & Tatler, 2009). Some individuals choose to develop these processes into a form of perceptual–cognitive expertise. The study of individuals who have through years of training honed their neural mechanisms to the high state of tune needed to carry out these tasks therefore allows researchers an opportunity to observe perceptual–cognitive expertise at the limit.

The purpose of the paper is to present a case study in the classic sense by describing a paradigmatic example and how to dissect or diagnose it. Our “case” is the visual strategy of an expert racing driver, as an exemplar of visuomotor coordination in the wild, and the analysis involves the concept of developing an integrated dataset that can be cross-examined with different parametrizations in order to address different types of research questions. We believe this is a generally useful and interesting methodological approach to complex, multisensor, time series and localization data.

We have organized the methods and results around four general classes of research questions: (a) oculomotor, (b) scene analysis, (c) timing, and (d) point of vantage. We are not aware of any study that has simultaneously addressed all four kinds of research questions using naturalistic gaze data from a single extended behavioral sequence. We also highlight particularly the last kind—investigating the strategic use of points of vantage—as a distinct paradigm (combination of research questions and analysis concepts). It only makes sense in mobile contexts and, perhaps because of that, is much more rarely posed or recognized as such.

To anticipate the results themselves, we (a) replicated the seminal tangent point orientation (pre–turn-in saccades) and “steering with the head” strategy, (b) identified a novel saccade strategy (pre–full-throttle saccades/exit fixations), and (c) revealed previously unstudied spatial regularity in the serial organization of behavior, a tight localization of points of vantage for specific gaze behaviors. This integrative methodological approach allowed us to examine all of these.

The problem we address—expert performance in high-speed sports and the role of specific gaze strategies within that—is not specific to the task and is equally relevant for expert performance in other fields with similar visuomotor and cognitive demands. Of course, to what extent the specific visual strategies observed here generalize across other modes of locomotion available to humans (e.g., skiing, surfing, skateboarding; bi- and quadrupedal locomotion in acrobatics, gymnastics, or parkour) requires detailed study of each behavior, in both controlled and naturalistic contexts. Only this type of analysis of natural gaze strategies can, eventually, differentiate between truly general principles and more task-specific cues and techniques.

## Methods

**Figure 1.**
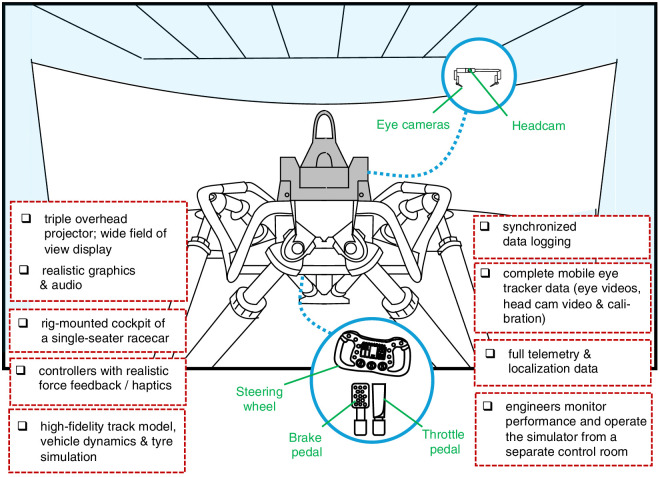
Simulator setup. Schematic depiction of the experimental apparatus. A racecar chassis (gray) is mounted on a hydraulic moving platform in front of a large field-of-view projector screen. The steering wheel (with gear-shift paddles), throttle, and brake (inset) are used to control the simulation. A mobile eye tracker (inset) was used to measure eye movements and head pose. (For photographs and a video, see https://www.avlracetech.com/race-engineering/.)

**Figure 2.**
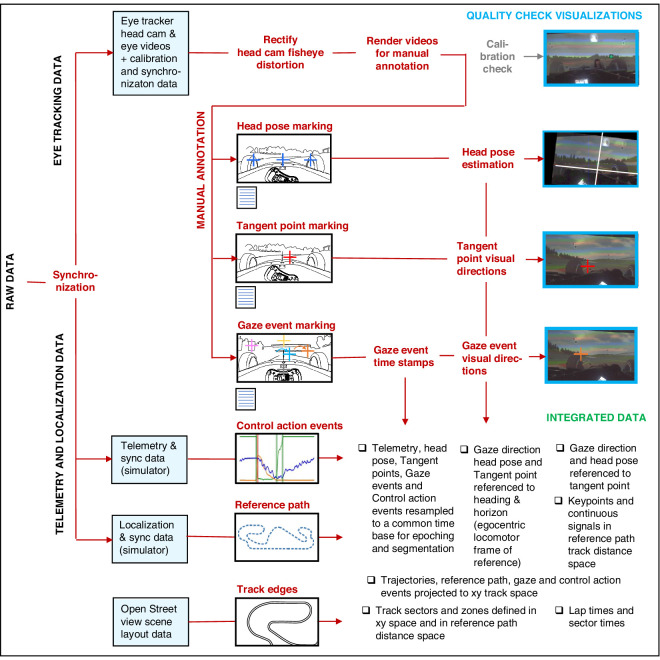
Analysis concept. To obtain an integrated dataset, the workflow combined telemetry, gaze, and localization information. This provided analysis flexibility to address various research questions (different parametrizations, multiple phenomena) in a single behavioral sequence.

The principal strengths of this paper are the use of a high-fidelity simulation platform (Figure 1) and the custom analysis workflow that combined gaze, telemetry, and localization data (Figure 2). The latter in particular enables the kind of flexible parametrization that allows us to address the multiple types of research question that we desire. The main text describes methodology at a level of detail necessary to understand the results. More detail intended for those who wish to replicate our design is found in the Supplementary Methods and Results. Both raw and postprocessed data and the code for the visualizations and analyses are available through the Open Science Framework (https://osf.io/qcu7t/).

### Participant, equipment, and experimental task

The driver was a 24-year-old professional male racing driver. He had started to race at the age of 6 and had been competing at the international level since the age of 14, with experience in single-seater and GT racing cars (e.g., BOSS GP Racing Series; ADAC GT Masters; GP3 Series; Euroformula Open Championship; ATS F3 Cup; Austria Formula 3 Cup). Informed consent was obtained, and the tenets of the Declaration of Helsinki were followed.

The simulator rig was a high-grade professional driving simulator used for driver-in-the-loop vehicle simulation, typically for research and development purposes at the global motorsport division of AVL List GmbH (Graz, Austria). The system is used by top-level motorsports engineering companies to perform driver-in-the loop component testing, and we deemed it to represent industry gold standard at this time (Figure 1). A steering wheel with realistic force feedback and a brake pedal with a high spring rate simulate the feel and the ranges of limb movement and force magnitudes required to control a real racing car. The controllers were fixed into a single-seater cockpit mounted on a motion platform (the motion functionality was not used in this experiment). The simulator was running AVL VSM and FASTLAP simulation software, and the graphics were rendered using a triple DLP projector system on a large curved screen (approximately 180° field of view, with a viewing distance of about 3 meters). For more details, see Remonda, Veas, and Luzhnica (2021).

The Circuit de Barcelona-Catalunya and a Formula 2 car were simulated. The track length in this configuration is 4.675 km (14 turns). The elevation changes 29.6 meters from the lowest to highest point, and the track width is about 12 meters. The car is a high-powered, high-downforce race car requiring expert skill to handle and extract consistent lap times. Lap times in the 1 minute 30+-second range yield nominal average speeds in the 180-kph range. Top speeds in excess of 270 kph were achieved on the main straight.

The Pupil Core mobile eye tracker (Pupil Labs GmbH, Berlin, Germany) was used to measure eye movements. Eye cameras were set to run at 60 fps, scene camera at 30 fps. The natural features calibration functionality of the pupil capture software was used to calibrate eye-in-head direction by presenting a physical fixation target at successive locations. Verification of the calibration was done before proceeding to the test run. Visual inspection of the gaze overlay video (see below) indicated good calibration, with no major bias from headset slippage or data loss as assessed by inspecting the exported gaze overlay headcam videos.

The experimental data used for this study included a driving session (stint) of 15 laps of dry-weather running. The driver was asked to drive at the maximal speed he could sustain without crashing. Because of the high level of expertise of our participant, he was able to perform this task without crashing, which is a common problem leading to data loss when this instruction is given to non-elite drivers (van Leeuwen et al., 2017; Vogel et al., 2022a; Vogel et al., 2022b; Joyce, Campbell, Hojaji, & Toth, 2024). The experimental task was a basic race driving task: lapping the circuit without other cars present. This allowed pinpointing the driving skill determinants, as opposed to racecraft skills such as attacking or defending against opponents (strategic decisions).

The present data are part of a larger dataset (187 laps, 14 stints on two different race tracks), where the driver drove in various conditions (dry, wet, sometimes under a cognitive load manipulation). The stint we analyzed was collected in the middle of the first testing day, so the driver would have been thoroughly familiar with the car and the track, based on his morning runs. A much larger set of physiological measures (e.g., electromyogram, electrocardiogram, electroencephalogram) were also collected in the session; however, these other physiological measures are not considered in this paper.

### Data preprocessing, signal analysis, and annotation

#### Synchronization

Eye-tracker data time stamps, head-camera (head-cam) video, and simulator telemetry/localization were all time stamped based on the operating system clock and thus shared a common time base. This was a critical step for all subsequent analyses.

#### Telemetry data

The simulation software provided detailed vehicle systems and dynamics data of several hundred parameters, as well as timing data in the form of lap time every time the car crossed the start/finish line. Lap time is the major criterion performance metric for a racing driver. Because of the small number of laps and high consistency (i.e., low variability in performance), we did not analyze performance variability between laps. The other signals afford a detailed analysis of expert performance. Here, signals from the steering, brake, and throttle (gas pedal; see Figure 1) were used to segment the telemetry data.

**Figure 3.**
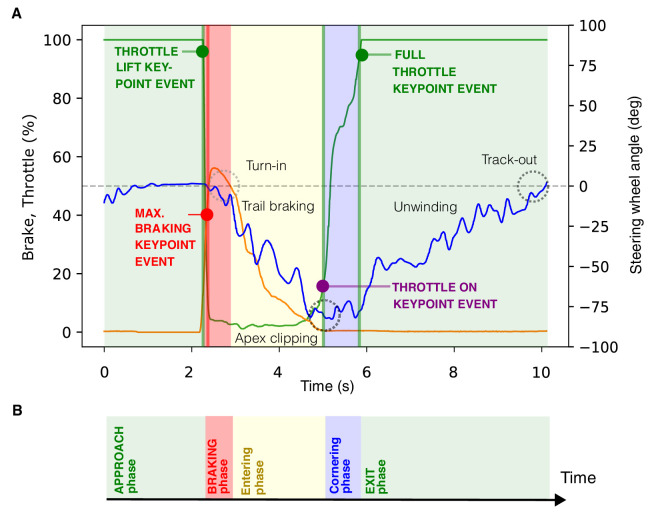
Control action keypoint events used for epoching the cornering sequence into phases in the time domain. Discrete actions performed on the controllers segmented the telemetry into approach, braking, entry, cornering, and exit phases. (**A**) A sample of telemetry time series data (the fastest run through turn T4). Vertical lines indicate algorithmically detected control action keypoint events. Manual labeling of other driver actions and outcomes is given for context. Shading indicates the phases. (**B**) The racing line partitioned into a sequence of phases in the time domain. Blue indicates the steering wheel angle; green indicates the throttle position; orange indicates the brake pedal position. Control action events were defined as follows: throttle lift, from maximum throttle pedal position to zero (event detection threshold < 95%); maximum braking, from zero to maximum brake pedal displacement (event detection threshold > 40%); and full throttle, accelerator pedal position reaches maximum value (event detection threshold > 95%).

We identified discrete control actions in the telemetry and refer to them as control action keypoint events when they are used used as epoching events to partition telemetry time series data into phases (Figure 3). The partitioning was done as follows: The *approach phase* is characterized by approaching a corner with the gas pedal depressed fully to 100% (i.e., on full throttle), zero brake pedal pressure, and centered steering. It lasts until the throttle lift event, where the throttle moves almost instantaneously from fully depressed (100%) to fully off (0%), and a maximum braking event (high brake pedal pressure, close to the maximum braking capability of the vehicle is applied). These events mark the start of the *braking phase*, which blends into corner entry phase as the steering wheel is rotated from the straight-ahead position (turn-in) while the brake is gradually released (“trailed”). The beginning of the *cornering phase* is marked by a throttle-on event (the throttle is reapplied above some threshold value indicating neutral throttle). The *exit phase* begins with the first full throttle event (the throttle pedal value reaches 100% again), during which the steering angle returns to straight ahead (“unwinding” the car to track out).

This is the basic form of a meaningful cornering sequence in a geometrically simple bend between two straights (Lopez, 1997). Negotiating connected bends and more complex surface geometry, especially in the *z* (vertical) direction, would add complexity and variations to this basic theme (e.g., richer and more nuanced selection of [sub]actions could be used to define more control action keypoint events and [sub]phases). For the present work, this sequencing framework offers sufficient detail for analysis. Also, because the driver was a professional-level expert, the observed behavior is very consistent and there was no data loss from failure of the telemetry to fit this parametrization framework. For novice drivers with more inconsistent and difficult to parametrize behavior, greater flexibility and complexity might be required.

#### Localization data

The simulation software provides a ground-truth localization of the car on the track (in world *x*, *y*, *z* coordinates), as well as track distance traveled (in meters; reset to 0 at every lap). The time series of different laps will have different numbers of observations depending on the duration of that lap. To compare multiple runs, the standard approach in performance engineering is to visualize time series data resampled to distance covered, as this allows actions and events at the same track location to be overlaid for inspection. (Thus, in presenting the data as distance series rather than time series we are looking at gaze data much as a performance engineer would look at car telemetry data) (Fey, 1993; Segers, 2014). Such a track distance coordinate system reduces the four-dimensional (*x*, *y*, *z*, *t*) observations into a single dimension.

**Figure 4.**
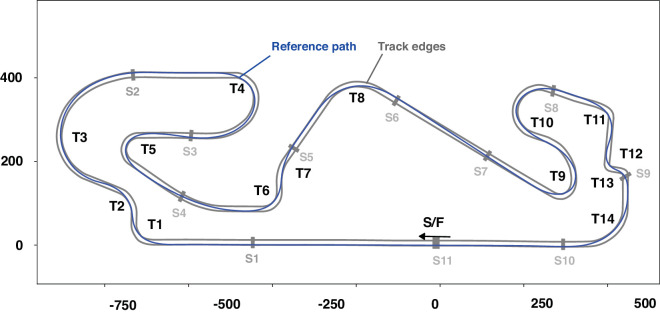
Track map. The Barcelona circuit layout used and the reference path. T1 to T14 designate numbering convention for the curves, S1 to S11 (the numbering of sectors or reference locations for “timing splits”). The solid blue line indicates the reference path used to convert time series and *x*,*y* localization data to track distance values (the racing line, represented as successive locations on the plane of travel). The scale of the *x*-axis and *y*-axis is in meters. T4, T5, T8, T9, T10, and T11 are simple bends, whereas T1, T2, T3, T6, T7, T12, T13, and T14 are more complex sequences where each bend is heavily influenced by the preceding/succeeding bends.

A commonly used distance measure is based on path integration (which we refer to as path distance or odometer distance). This measures the actual trajectory taken by the vehicle on each run. However, because of variability among runs in the exact trajectory taken, this measure accumulates between-runs error variances (largest at the end of the lap). Preliminary analysis indicated considerable variability, even with a consistent expert driver (see Supplementary Methods and Results). We therefore defined a track distance measure based on a common reference path as the average of successive odometer readings on the *x*,*y* plane of travel, ignoring the (vertical) *z* coordinate. Track edgelines allow defining the track as a surface in *x*,*y* space, providing a useful bird’s-eye view. Coordinates for the actual Circuit de Barcelona-Catalunya were recovered from Open Street view and matched to the reference path. (Figure 4). We refer to successive positions when projected onto this path as reference path distances.

The discrete control action keypoint events used to define phases in the cornering sequence can be associated with a location (both in *x*,*y* space and on the reference path). We refer to such locations as control action keypoint locations. Based on the reduction of the world (*x*,*y*,*z*) or plane of travel (*x*,*y*) into a single reference path distance coordinate system for the track space, we can use locomotor control action keypoint locations to sequence the racing line into track segments.

**Figure 5.**
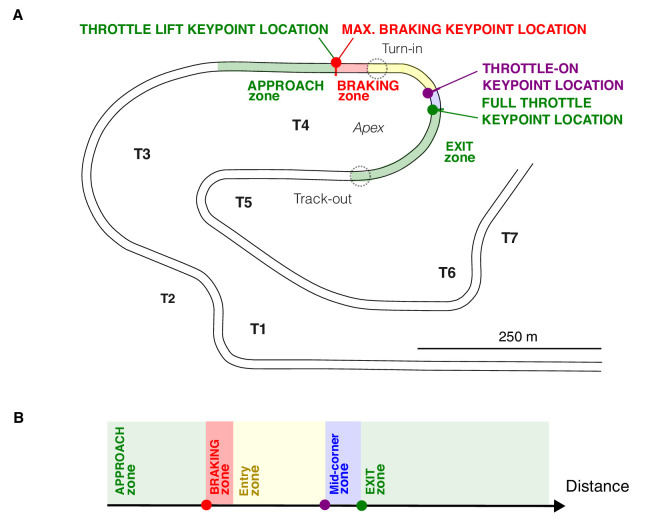
Control action keypoint locations and corresponding zones in space. Control action keypoint events (throttle lift, maximum braking, throttle-on, full throttle) can be projected into control action keypoint locations in track space (solid dots). When the control action keypoint locations of different laps align, the track can be partitioned into geographically fixed zones. Zones are thus defined as track segments where control actions of a specific type occur and will generally contain all control action keypoint locations of that type. (For all of the observed keypoints in track space, see Figure 12). (**A**) Approach, braking, cornering, and exit zones for turn T4. Track space is represented in the *x*,*y* plane of travel. (**B**) The sequence of zones in the path distance domain segmenting the localization data in a similar way as phases of the cornering sequence used to segment time domain data.

These segments for different laps do not overlap exactly if the driver performs the relevant actions at slightly different locations. The high consistency of the expert driver, however, allows us to partition the reference path into sectors fixed in space—what we refer to as approach, braking, entry, cornering, and exit zones*.* We define the braking zone of each corner to begin at the first observed throttle lift keypoint location and end at the last throttle lift keypoint location. In other words, all braking across all of the laps always happens in this zone. The mid-corner zone begins at the first throttle-on control action keypoint location. The exit zone begins at the first full throttle keypoint location and ends at the next sector boundary. Figure 5 illustrates the concept (zones for turn T4 are shown, with the zone-defining earliest lift, throttle-on, and full throttle keypoint locations indicated).

#### Eye-tracking data

The mobile eye tracker provides video data (forward-looking head-cam video and eye video) and calibration data for determining the direction of gaze in the head coordinate system. These can be analyzed to identify gaze targets and gaze direction relative to important scene elements and to determine movement of the eye (and the head) in different coordinate systems. Synchronizing eye-tracking data with the telemetry and localization data moreover allows investigating the timing of gaze events relative to locomotor action events and where these events occurred in the allocentric locomotor space.

##### Head pose marking

**Figure 6.**
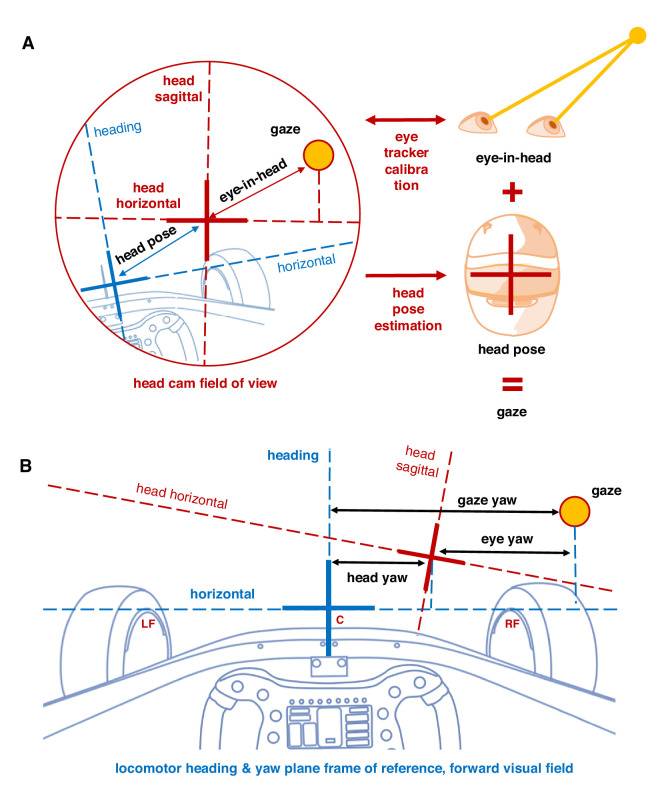
Coordinate transformations. Schematic explanation of frames of reference referred to in the Methods and Results sections. (**A**) Head frame of reference. The native coordinate system of a head-mounted eye tracker after gaze calibration provides eye-in-head gaze values only, corresponding to gaze direction in the head-cam field of view. When combined with head pose estimation, we obtain gaze direction in a heading (direction of travel, straight ahead visual direction)/horizon (plane of travel, yaw plane) frame of reference. Unless indicated otherwise, visual direction and eye/head/gaze rotation (yaw) is reported with the heading as reference and the displacement measured along the horizontal. (**B**) Head pose and gaze direction in a locomotor (heading, car, display) frame of reference. We generally describe both head and gaze horizontal rotations (i.e., yaw) relative to heading (i.e., this forward visual field). We define the eye-in-head horizontal direction in this coordinate system as gaze yaw – head yaw.

A head-mounted eye tracker, when calibrated, provides only an eye-movement signal relative to the head. In order to determine gaze direction (i.e., rotation of the line of sight) relative to heading, it is necessary to determine the head pose in a locomotor frame of reference, which in our setup was defined at rest relative to the display center/body centerline and (virtual) vehicle longitudinal axis. (These coordinate systems all represent the same frame of reference.) This reference frame was established by manually annotating from the head-cam video tops of the left and right wheel rim, as stable reference frame features (0.25-second intervals). These define the horizontal or yaw axis of the visual field. The aerial antenna on the (virtual) chassis midline was annotated and used to represent the heading or straight-ahead direction (note that this differs from the actual velocity vector by the amount of vehicle yaw slip). These were interpolated to determine the vehicle-to-head and coordinate transformation at each time point, the eye-in-head signal then yielding gaze in space. These are defined as shown in Figure 6.

##### Gaze event and tangent point marking

It is often convenient to express gaze and head direction relative to the tangent point, the tip of the curve apex in the visual field.^2^ Referencing gaze to the tangent point allows aggregating gaze direction observations over time and across different bends into a common coordinate system that removes variability in head pose and vehicle heading. Tangent points of each run through each bend were manually annotated from the head-cam video using a custom frame-by-frame annotation tool (at 0.25-second frame intervals) and from a video synchronized to the telemetry and localization data and interpolated and resampled to yield a tangent point signal.

**Figure 7.**
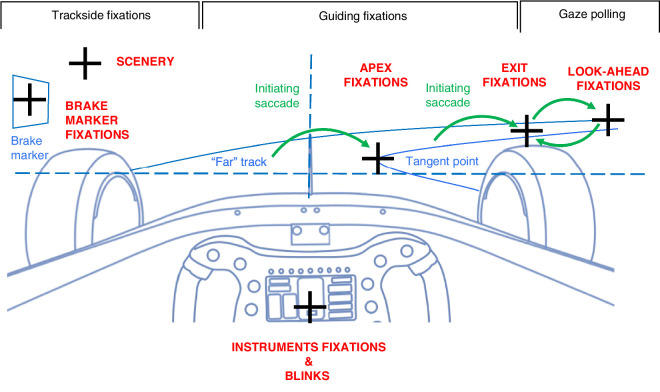
Gaze event and scene target marking used in the visual scene analysis. *Apex fixation*: The final saccade toward the curve apex before entering the bend initiates apex fixations. This saccade lands on or near the tangent point, followed by one or more fixations along the inner road edge (and especially in slower bend optokinetic nystagmus). Initiation of the apex fixation sequence was marked. *Exit fixation*: Before leaving the bend, a large saccade toward the curve exit was followed by guiding fixations on the far track. Initiation of the exit fixation sequence was marked. *Tangent point*: The tip of the curve apex is shown in the visual field projection as a travel point on the inside track edge moving with the observer. It is only present in bends. *Instruments*: Dashboard display on the steering wheel and RPM indicator shift lights. *Blinks*: Complete closures of the eye lid and may coincide with gaze shift. *Brake marker fixations*: At the end of the straights (approach to turns T1, T4, and T9) there were numbered boards at the side of the track indicating distance to the next bend. The rest of the time gaze occurs mainly in the far road region (guiding fixations), with occasional gaze polling saccades further along the bend and back (look-ahead fixations). Glances at the peripheral scenery are rare.

The initiation of a fixation sequence (glance) was likewise identified manually from the head-cam video by frame-by-frame analysis (0.033-second frame intervals). Five classes of these gaze events were identified: (a) initiation of apex fixations by a saccade toward the tangent point on the curve apex, (b) initiation of exit fixations by a large saccade away from the tangent point, (c) brake marker fixations, (d) instrument fixation, and (e) blinks (Figure 7). Synchronization to localization and telemetry data allowed us to assign a time stamp and a track location to each event.

##### Timing and localization analyses

Gaze–action timing was analyzed in two ways: (a) cross-correlating the gaze direction and steering time series, and (b) using an event-based analysis to associate task-relevant saccades identified in scene analysis with control action events identified in telemetry analysis. For the latter, gaze data involve discrete events (saccades, blinks) that can be identified as gaze events. Gaze events identified in scene analysis have an associated plane of travel (*x*,*y*) and track distance location, which we refer to as gaze keypoint locations. They represent the observer point of vantage at the time of the gaze event. These gaze keypoint locations can be used to segment spatial data or define zones on the reference path analogous to control action keypoint locations above.

##### Visualization of integrated data

For Supplementary Movie S1, all observations were matched based on their successive track distance positions; that is, observations with the same reference path distance value were rendered in the same frame, overlaid on a head-cam image of one lap (all observations transformed to the locomotor frame of reference).

## Results

Where does the expert look? How are eye and head movements coordinated? How is this coordination timed with respect to locomotor actions? Where are specific gaze behaviors performed? The flexible custom analysis workflow allows us to examine this complex dataset from multiple points of view—each of these four types of research questions. Here, the results are organized accordingly, with each section addressing a different set of problems by parametrizing the data in different ways:

• Gaze targets in the visual scene (Scene analysis results)
• Eye–head coordination (Oculomotor results)
• Coordination between gaze and locomotor control (Timing results)
• Localization of gaze behaviors in allocentric locomotor space (Point of vantage results)

Taken together, they present a fairly complete picture of the complex dynamic behavior of a single extended sequence (over 20 minutes) of skilled behavior, serving as a case study of the visual strategy of an expert racing driver embedded in the unfolding flow of locomotor coordination.

**Figure 8.**
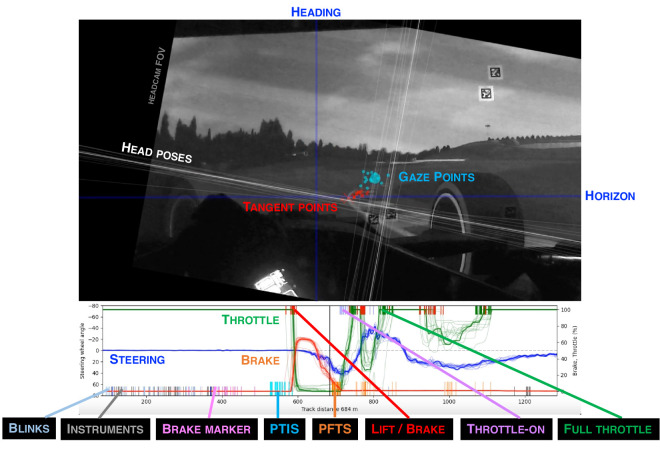
Guide to Supplementary Movie S1 (https://osf.io/nze3p). The movie is a composite of multiple laps. This allows visualization of both temporal spatial consistency of the behavior across different laps in a way that a gaze overlay video frame or time series showing only one observation at a time would not. (Left) Example frame. Note that the ambient lighting level in the simulator was low, and that there is some rotating lens projector (DLP) rainbow pattern artifact visible on the video that was not visible to the human eye. The black vertical line indicates heading, and the black horizontal line indicates horizon. White cross hairs indicate head pose, yaw pitch, and roll at this track location on each of the 15 laps. Round dots indicate gaze direction in the egocentric locomotor frame of reference on each of the 15 laps. They are color coded according to gaze event type (see main text): cyan, pre–turn-in saccade followed by apex fixations; orange, pre–full-throttle saccade followed by exit fixations; gray, instruments; pink, brake marker. Red crosses indicate tangent point visual direction (i.e., relative to heading) at that track location on each of the 15 laps (the variation comes from slight differences in racing line on different laps). Brake, throttle, and steering wheel angle signals and the gaze and control action keypoints, all resampled to track distance, are shown at the bottom. The thick line is the median value of the signal at the given location, and the thin lines in the background are the signals of each individual lap. The black vertical line indicates the track location of the current frame. The red and green vertical dashes indicate lift/brake and full throttle events, respectively. The cyan and orange vertical dashes indicate pre–turn-in-saccade and pre–full throttle saccade events, respectively, preceding these control events (see Timing results and Point of vantage results sections). None of the annotation overlay visualizations was visible to the participant.

To first get an overall feel for gaze-control action coordination in time and space, the reader may wish have a look at the Supplementary Movie S1 (https://osf.io/nze3p). Because of the high-speed nature of the task, we recommend slow-motion or frame-by-frame viewing, cross-referencing to the key in Figure 8 as needed.

### Scene analysis results

Perhaps the most basic question examined in eye tracking data is “Where is the subject looking?” The most salient observation is that, before entering each turn, the gaze makes a clear saccade to the inside road edge, initiating the apex fixation sequence, wherein gaze remains locked to the curve apex for much of the curve. This saccade frequently lands on or near the tangent point. This observation has been made in all previous eye tracking studies of race driving and is sometimes referred to as tangent point orientation (Land & Tatler, 2001; van Leeuwen et al., 2017; Lima et al., 2020). However, it is only the initiation of the apex fixation sequence that is tangent point oriented, and not all of the apex fixations remain on the tangent point (much of the time they are directed well beyond the tangent point). In the bends, gaze is tightly focused or locked on the general visual direction of the apex, but this does not imply that gaze is stable or fixed on one point in space or in the visual field (especially in slower bends, some optokinetic nystagmus and gaze polling can be seen).

**Figure 9.**
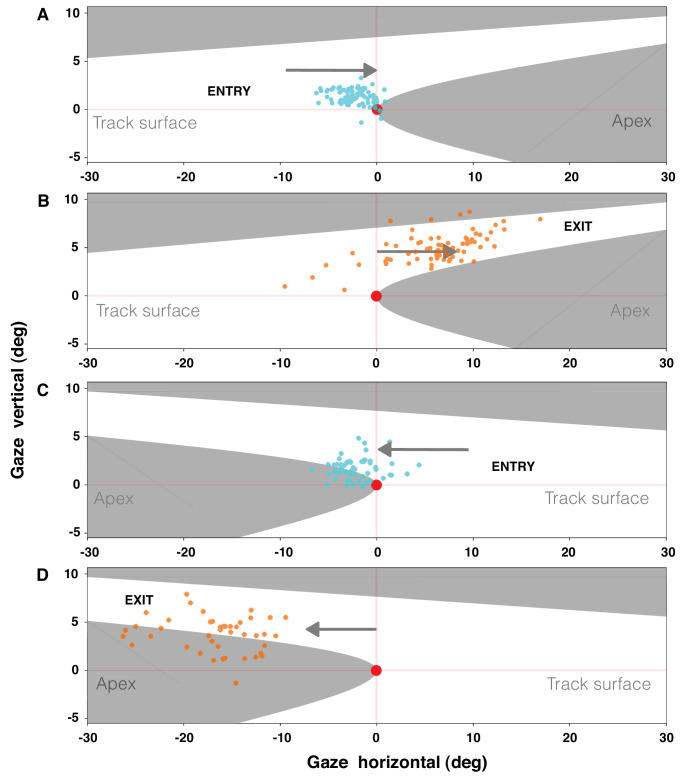
Scene analysis results showing initiation points of the apex fixation (cyan) and exit fixation (orange) sequences. Data for left-hand and right-hand bends are shown separately. Data are plotted relative to the tangent point (TP, the tip of the apex in the visual field) visual direction as the origin of the coordinate system. Track surface and apex are indicated schematically only (the exact shape the track present in the visual field varied slightly in different turns, depending on the exact curve geometry). (**A, C**) Apex fixations were initiated by a saccade toward the curve apex, at or close to the tangent point of the inner edge line, an oft-reported behavior in both race driving and everyday driving. (**B, D**) Exit fixations were initiated by a large saccade away from the tangent point (indicated by a gray arrow). This is a novel observation. Note that the coordinate system orientation was transformed from the eye-tracker coordinate system to the “level” locomotor coordinate horizontal (yaw plane) and vertical (heading).

A novel observation is that, once the car had taken a set into the bend, our driver also made at least one very prominent, large saccade before exiting corners. Some distance into the bend, his gaze was unlocked from the apex. This saccade was made away from the tangent point and initiated exit fixations, which blended into the guiding fixations (GFs) on the following straight. Figure 9 shows the initiation points (i.e., saccade landing points) for both the apex fixation sequence and the exit fixation sequence.

One notable (negative) observation is the absence of visual search or scanning of the the scenery—out of 840 identified gaze events, only 12 were to unspecified targets in the trackside scenery (1.4%; see Supplementary Event Coordinates Data). On the straights, gaze was in the straight-ahead direction, locked to the far-road GF region. Saccades away from the track were almost always either to the visual display/control buttons located on the steering wheel or to brake markers at the side of the track (posted signs at the ends of straights indicating distance to the next bend). Gaze polling (Wilkie, Wann, & Allison, 2008) of the curve exit or the upcoming turn with look-ahead fixations (LAFs) can sometimes be seen.

### Oculomotor results

The second most basic question is “How does the eye move?” In head-free viewing, the rotation gaze direction in space is achieved by combining eye-in-head movement to head rotation; that is, the movement of the eye is achieved in part by the head (Land, 2006; Land & Tatler, 2009). (Oculomotor gaze coordination must therefore always take into account both eye calibration and head pose estimation.)

**Figure 10.**
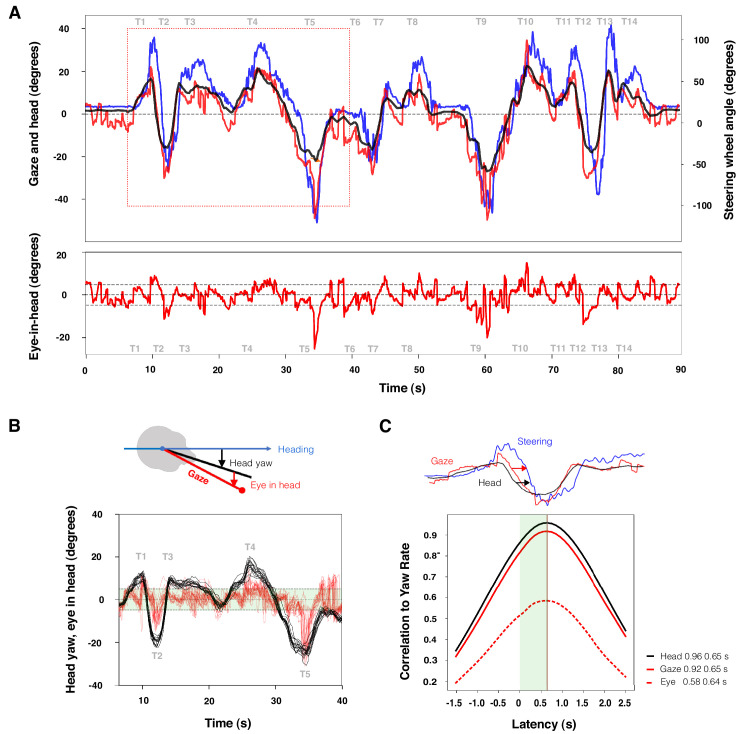
Oculomotor and timing analysis results. (**A**) Head, gaze, and steering wheel angle (black, red, and blue, respectively; top panel) and eye-in-head (red; bottom panel) time series for one lap. Dashed horizontal lines indicate ±5° of eye-in-head. The red rectangle indicates selection in the panel. (**B**) Oculomotor signature of steering with the head. Large head movements followed the rhythm of the track established by the curve geometry and anticipated steering; eye-in-head movement is smaller amplitude and did not follow the low-frequency rhythmic pattern (all 15 laps for turns T1 to T3 are shown). (**C**) Timing signature of steering with the head. Time series cross-correlations for head yaw direction and locomotor yaw rate (black); gaze yaw direction and locomotor yaw rate (solid red); and eye-in-head yaw direction and locomotor yaw rate (dashed red). The cross-correlation for head is of much higher magnitude than for eye-in-head.

Figure 10A plots head pose (in the yaw plane, with head rotation relative to heading), and eye-in-head (defined here as horizontal gaze yaw, or head yaw in the locomotor coordinate system). The figure shows the way in which large rotations of the head followed the rhythm of the race track, with residual eye-in-head rotation not synchronized to the large-scale curve geometry. Most of this residue is small amplitude (within about 5° of head direction), but there are some notable peaks.

This pattern was first reported by by Land and Tatler (2001): “It is the head that performs most of the required movements, while the eyes stay within about 5° of the head axis for most of the time.” This is one of the characteristic features of the eye–head–steering coordination pattern, which they dubbed “steering with the head.” We refer to it as the oculomotor signature of steering with the head (Figure 10B).

### Timing results

The third question is “How are gaze and locomotion coordinated in time?” Gaze leads steering (which can be defined as vehicle rotational rate, steering wheel angle or lateral position relative to a reference path). That this anticipatory rotation is dominated by head movement, rather than eye-in-head movement is the timing signature of steering with the head (Figure 10C).

Land and Tatler (2001) quantified this by a high cross-correlation (0.94) between head yaw angle and vehicle yaw rate with a lag of 0.9 second. Gaze–yaw rate correlation was slightly lower (0.9), and eye-in-head–yaw rate correlation was a mere 0.08. In other words, it was head–hand coordination more so than eye–hand coordination that established the rhythm of the racetrack. We replicated this analysis in our data and found almost identical head–yaw rate and gaze–yaw rate cross-correlation magnitudes (0.96 and 0.92, respectively). The lag value of cross-correlation peaks was shorter, 0.65 second. (This likely reflects a difference in the dynamics of human–machine interaction in the two experiments: the Land and Tatler experiment was conducted on a wet track in a contemporary F3 car, whereas our experiment simulated a more modern F2 car, with much higher achievable rotational accelerations, or sharper, pointier responses.)

In our data, the eye-in-head cross-correlation, although considerably lower than eye or gaze cross-correlation values, is considerably higher than theirs, at 0.58. This might reflect an individual difference in visual strategy or, more likely, differences in track conditions: On a wet track, their driver may not have been able to use his usual braking point references, leading to more scanning behavior to select and fixate at new markers reflecting the longer braking distances in the low-grip conditions. Not too much should be read into this, however, as signal cross-correlation is a coarse-grained analysis, especially when it is done across the entire dataset, including both the bends, where gaze is locked to the curve apex and thus highly correlated with steering, and the straights, where gaze is free to scan but steering must be kept straight. This leads to the implicit statistical assumption that the signals are driven by a single underlying generating distribution or process (across the whole lap) being dubious.

### Point of vantage results

The fourth question is “Where in space are gaze actions performed?” From the oculomotor data we see that, much of the time, gaze and head are aligned. On the other hand, there are substantial peaks and discontinuities in the eye-in-head angle. From the scene data, we can moreover see that large saccades initiated both apex and exit fixation sequences. Identification of discrete control action and gaze events and the localization of these events in track space as keypoint locations allows us to investigate this behavior from the point of view of spatial regularity.

**Figure 11.**
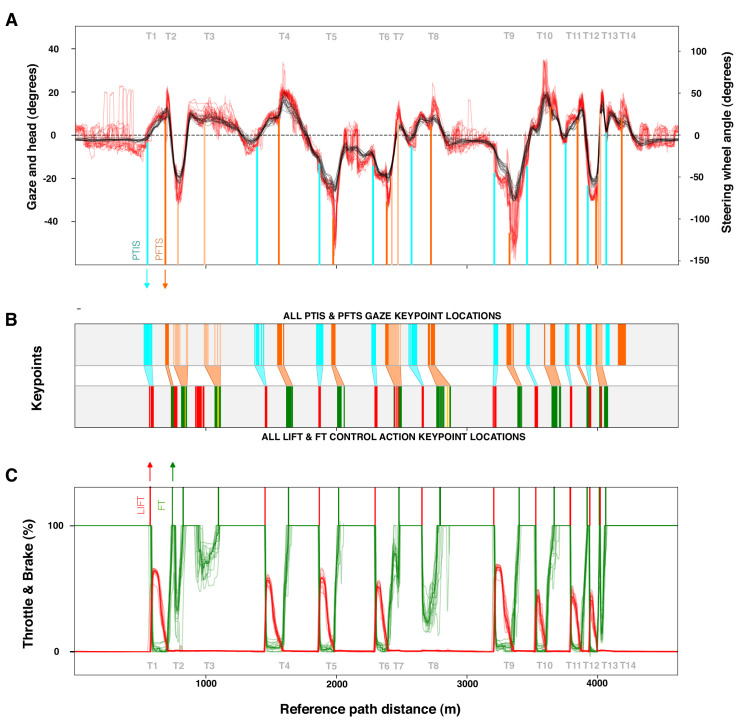
Behavioral consistency and sequential organization of keypoint locations. (**A**) Distance series of gaze (red) and head pose (black), all 15 laps overlaid. Cyan and orange vertical lines indicate approximate track locations where pre-turn-in saccades (PTISs) and pre-full-throttle saccades (PFTSs), respectively, were made. Note the consistency of gaze behavior across laps (e.g., the PFTS peaks, indicated by vertical orange lines). (**B**) All PTIS (cyan) and PFTS (orange) gaze keypoint locations and throttle lift (red) and full throttle control action keypoint locations (orange) from the 15 laps. There is clear clustering or a spatial rhythm to both the gaze keypoint location distribution and control action keypoint location distribution. The sequences, moreover, seem to be in register with one another. The diagonal connectors suggest grouping of specific PTIS clusters with lift clusters and PFTS clusters (see Figure 12 for more detail). (**C**) Distance series of throttle (green) and brake (red) pedal positions, all 15 laps overlaid. Green and red vertical lines indicate approximate track locations where full throttle and lift to turn-in actions, respectively, were made. Note the consistency of control actions across laps.

Figure 11 shows two major classes of gaze events—pre–turn-in saccades (PTISs) and pre–full-throttle saccades (PFTSs)—in relation to two major decision points in the curve sequence: lifting the throttle to turn in and getting back on full throttle to track out. (Note that plotting the data in spatial coordinates allows overlaying the laps without drift that would happen if we plotted the data as time series.)

**Figure 12.**
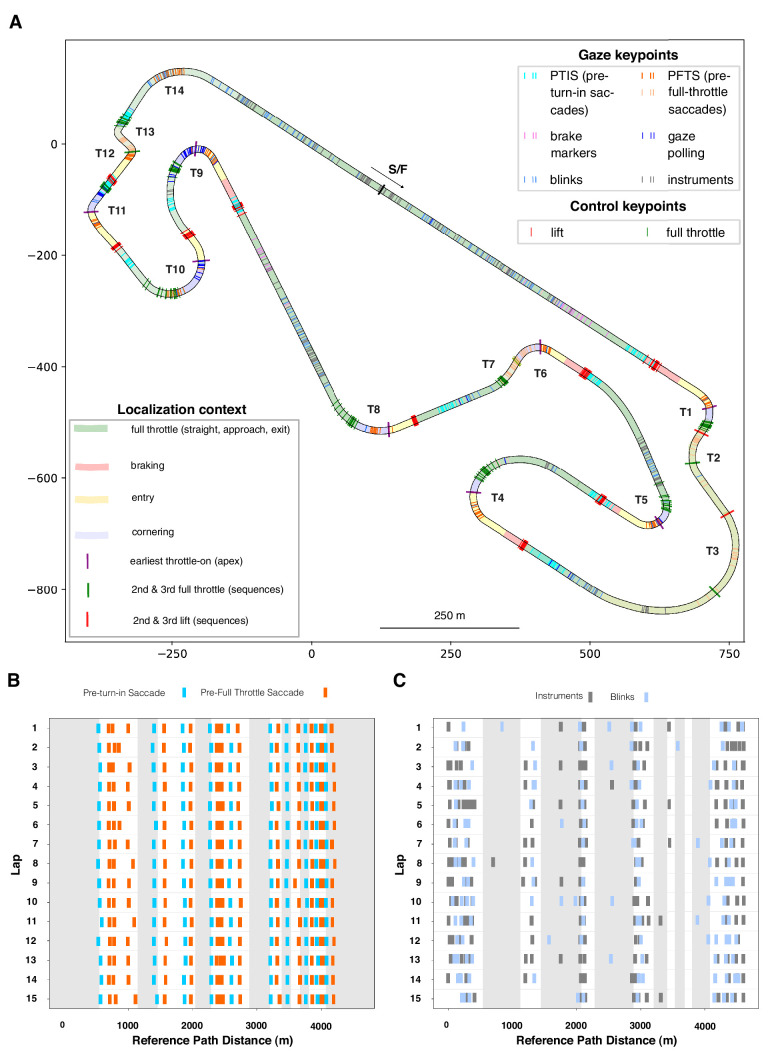
Individual gaze and control action keypoint localization. (**A**) Gaze and control action keypoint localization on the plane of travel, at the level of single fixations/saccades. The track is divided into segments based on control actions. Green shading indicates full throttle segments (straights, approach, and exit zones of the turns), red shading indicates braking zones, and purple shading indicates mid-corner zones. Yellow indicates the entry zone between braking and mid-corner (Figure 5). All gaze keypoints for pre–turn-in saccades (PTISs; cyan), pre–full-throttle saccades (PFTSs; orange), look-ahead fixation (dark blue), instrument fixation (gray), and blinks (light blue) are shown. Note the clustering of PTIS prior to the braking zones and PFTS prior to the exits, and blinks and instrument fixations mainly on the straights. (**B**) Raster plot of individual gaze keypoint locations against reference path distance, each lap (1–15) shown separately. Gray shading indicates straights, and white shading indicates the turns and turn sequences. Note the systematic serial organization of turns (or turn sequence) initiated by a PTIS and followed by a PFTS (multiple PFTSs in sequences), as in Figure 11B, but with each individual runs shown separately. (**C**) Raster plot of individual gaze keypoint locations against reference path distance, each lap (1–15) shown separately. Gray shading now indicates turns and sequences, and white shading now indicates the straights. Note the virtual absence of blinks and instrument fixations in the turns.

A noteworthy pattern is the clustering of the PTIS and PFTS gaze keypoint locations and the relationship between the gaze and control action series (Figure 11B). There is a spatial rhythm to both sequences, and they can be brought to register with one another across the whole track (indicated by the diagonal connectors to ease visual reference). The PTISs are associated with lift (and brake) events preparing the car for turn-in, whereas the PFTSs are associated with full-throttle events preparing the car for track-out. The PTIS keypoint locations and the PFTS keypoint locations can be considered to bookend an apex fixation zone on the track, of similar consistency to the entry and cornering zones on the racing line (Figure 12).

Figure 12 further illustrates this regularity in point of vantage localization at the level of individual fixations and saccades. Figure 12A gives a bird's-eye overview of all gaze keypoint locations on the plane of travel, embedded in their locomotor context (the straights and the different zones in the turns). Figures 12B and 12C further separate the PTIS and PFTS events of each lap and the blinks and instrument fixations of each lap, indicating lap-to-lap consistency across the 15 laps, demonstrating that this spatial organization indeed represents a systematic gaze strategy of the expert race driver.

## Discussion

A racing driver pushing the vehicle to the limits of its dynamic capability creates small time margins that leave little room for task-irrelevant gaze behavior. As the task demands are also pushing human information processing to its limits, performance likely depends on critically timed and spatially accurate gaze coordination.

We set out to systematically observe the skilled interaction of an expert racing driver in a domain representative task. We combined eye tracking with performance telemetry and localization data to deliver an integrated dataset that allowed us to investigate a single, extended sequence of expert performance (22 minutes). The analysis concept of an integrated dataset allowed different types of research question to be addressed via cross-examination through different parametrizations. We presented four types of analyses: four paradigms that we refer to as scene analysis, oculomotor analysis, timing analysis, and point of vantage analysis.

Scene analysis addresses the question “Where does the expert racing driver look?” by parametrizing the behavior in terms of the gaze point and gaze targets within the visual field. Here, we identified first of all tangent point orientation, or fixations, at the curve apex near but not quite at the tangent point. That drivers consistently make saccades toward the tangent point, to fixate the curve apex before entering bends, is a general and stereotypical characteristic familiar from prior literature (Land & Tatler, 2001; van Leeuwen et al., 2017; Lima et al., 2020) and broadly similar to the general pattern in road driving (Land & Lee, 1994; Lappi, Pekkanen, & Itkonen, 2013; for reviews, see Lappi, 2014; Lappi, 2022). Second, we presented as a novel observation that, before exiting bends, the driver makes another saccade, away from the tangent point and toward the curve exit.

We have also highlighted the absence of visual scanning or monitoring patterns, where gaze rotates broadly across the visual scene. Although ubiquitous in driving in traffic, especially for experienced drivers (Mourant & Rockwell, 1970; Mourant & Rockwell, 1972; Lappi, Rinkkala, Pekkanen, 2017), in race driving gaze is locked onto the track ahead. This is presumably because there are no traffic signs, no intersecting roads, or other road hazards typical of everyday traffic environment, as well as, in this case, no other track users (in a racing situation with opponents the situation would be different). Also, this suggests that, because the driver is highly familiar with the track, if any landmarks are used as visual reference for self-localization these are monitored almost entirely in peripheral vision. Any saccades away from the track are almost always either to the visual display and the control buttons located on the steering wheel or to visual brake markers at the side of the track, numerically indicating the distance to the upcoming corner.

Oculomotor analysis asks how the eye moves and is parametrized in terms of eye-in-head rotation (saccades, fixations), gaze rotation (eye plus head), and eyelid closure (blinks). Here, we highlighted especially the joint coordination of eye and head in the control of gaze (Land, 1992). In naturalistic tasks, pure eye movement (eye-in-head rotation) is of limited value, as gaze coordination is synergistic control of the eye and the head. The visual orientation toward the curve apex (identified through scene analysis) is achieved by a coordinated eye–head rotation marked by (midline-crossing) saccades in the direction of the bend (toward the tangent point) to initiate the apex fixation sequences. The oculomotor signature of the “steering with the head” strategy (Land & Tatler, 2001) was revealed by head movement dominating the gaze rotation, with a relatively lower amplitude residual eye-in-head rotation. However, a large (often double) saccade, directed away from the tangent point, was observed to initiate exit fixations sequences.

Timing analysis asks how eye movements are timed relative to other actions, and relevant parameters describe relationships between gaze time series and body (e.g., limb) time series, synchronized to one another. Inquiring into the temporal relationship between gaze coordination (eye–head synergy) and body movements (eye–hand coordination and locomotor synergies), we see that gaze leads steering, presumably picking up visual preview information to guide lateral and longitudinal control, but because of the oculomotor synergy this lead is achieved largely by head movement. This also belongs to the “steering with the head” strategy (Land & Tatler, 2001). The timing signature of the steering with the head was investigated by cross-correlating the gaze, head, and eye-in-head signal with vehicle yaw rate (approximately proportional to steering-wheel angle). Both head and gaze were found to be highly correlated (>0.9) with yaw rate at a peak latency at 0.65 second.

Point-of-vantage analysis asks where in space specific gaze behaviors are performed, and the parameters represent observer locations at the time of meaningful gaze behaviors and/or motor actions. In locomotor contexts, action is coordinated not only in time but also in space. Where a gaze shift happens can determine the subsequent visual input as much as (or more than) when it is done. But, although it is common to ask where a subject is looking—where the line of sight end point is in the scene—it is well to remember that the line of sight has two ends, with the other being the observer's point of vantage.

Gaze keypoint events and locations derived from joint analysis of eye tracking and localization data and control action keypoint events and locations derived from telemetry and localization data (providing a systematic task analysis of the context of the gaze behaviors) allow us to inquire into the organization of these points of vantage in space and their arrangement relative to locomotor actions and outcomes. Note that scene analysis does not in any way indicate these locations as “special” places where some salient visual feature would mark the location or suddenly pop into view. The consistency of localization (and timing) is rather a part of the expert driver's strategy or rhythm. Specific gaze behaviors are performed at specific points of vantage. Apex fixation sequences are initiated by pre–turn-in saccades in the approach and braking zones of each bend, anticipating turn-in. Exit fixation sequences are initiated by pre–full-throttle saccades performed when one is arriving at apex-clipping points preparing the driver to go to full throttle.

### Limitations

We investigated only four classes of control action keypoint events and locations (throttle lift, maximum braking, throttle on, and full throttle) and three classes of gaze keypoint events and locations (pre–turn-in saccades, pre–full-throttle saccades, and blinks). Other discrete locomotor actions and outcomes and gaze behaviors could be identified, and their keypoint locations should be explored.

With a case study, we can of course make statements only about our experimental subject. For other subjects, the results (e.g., concerning the reproducibility of the novel observation “spatial rhythm” of gaze keypoint locations) might differ, especially drivers of different levels of ability. Generally, we would expect weaker drivers to be much less consistent in their use of gaze compared with experts, as they are much less consistent in their use of controls. The high level of consistency in driving performance of our expert subject also allows for very crisp definition of the different phases and zones; less skilled and consistent driver behavior is more challenging to parametrize. Also, different, more geometrically complex track environments than the simple bends analyzed here may require additional steps in the parametrization of the relevant behavior.

This being a simulator study, the points of vantage only changed in virtual space (locomotor control actions on the controllers happened in both real and virtual space). The physical forces experienced by a subject in a simulator versus real world are different, which might lead to some changes in behavior or physiology. The correspondence of the gaze strategy to real-world race driving (sim to real transfer) should be established empirically.

The major physical difference is the constant gravitoinertial force (absence of variability in g-forces) and the absence of significant risk of injury in a simulation. On the other hand, a large field of view optic flow produces strong vection (but no binocular disparity cues), and in a high-fidelity simulation the way the environment responds to actions (changes in the simulated locomotor state) can be perceived through visual preview and haptic and proprioceptive feedback and controlled accurately and with precision. Similar, if not identical, perceptual–cognitive skills for locomotor guidance and control are, in other words, engaged (and transfer from real-world expertise to simulators and vice versa).

## Conclusions

Research on expert performance has shown that gaze coordination is a key feature in expertise, supporting exceptional visuomotor, attentional, and memory abilities with specific and replicable gaze strategies. In many fields of expert performance, including sports, investigating the gaze strategies of highly skilled individuals has proven to be a useful tool to probe the underlying perceptual–cognitive processes (e.g., Williams & Ericsson, 2005; Land & Tatler, 2009; Yarrow et al., 2009; Vickers, 2016).

What are the visual strategies of the high-speed sports expert, what roles do they play in guiding the unfolding action sequence, and what is the best way to study them? Eye tracking a high-performing athlete while they are engaging in stereotypical and repeatable, yet ecologically representative and challenging, performances offers an attractive model system for studying these problems, which may have implications for many outstanding questions in integrative brain function, such as the capacity of the brain to encode 3D scene information, to transform it into a coherent perception and memory representations, and to deploy these in executing well-coordinated motor actions (Land & Furneaux, 1997; Land & Tatler, 2009; Lappi, 2016). Although demonstrated here in the context of steering a wheeled vehicle, the analyzed gaze–locomotor coordination patterns are not specific and could be equally investigated in any other high-speed sport and other locomotor activities.

Field experiments and/or recreating domain representative tasks in simulators allow the skills and mechanisms to be deployed in their natural ecological context. This kind of study has an essential role in complementing laboratory studies on more restricted tasks. Before we can bring expert visual strategies into the laboratory for study and analysis, we must find out by measurement what the actual wild-type visual strategies are like.

In this pursuit, we believe the kind of cross-examination of complex behavior though different parametrizations is especially valuable in gaining insight into the integration of perceptual–cognitive and motor processes and is conducive to revealing in detail the richness of genuine slabs of human behavior (Newell, 1973).The most general takeaway from the present paper, therefore, is the way it demonstrates complex naturalistic behavior simultaneously parametrized in a number of ways to address multiple theoretically related research questions. Specifically, oculomotor, scene, timing, and point of vantage analyses and findings are all distinct and reveal different aspects of gaze strategy. The main methodological novelty of our study is the promotion of point of vantage analyses as their own, complementary set of research questions and the methodologies to address them—a fourth paradigm for studying gaze behavior in the wild, if you will.

In sum, our approach suggests a unique (perhaps even unorthodox) angle on naturalistic eye-movement behavior. Instead of devising an experiment with specific task constraints and instruction to probe a specific a priori chosen phenomenon, we asked the expert to perform a domain representative task in whatever way felt most natural to him, and we measured his behavior. Then, instead of comparing trials with different conditions and looking for statistical effects, we used different parametrizations of the complex data to address different research questions. We believe there is value in this approach to naturalistic behavior and that a clearer distinction among the different paradigms (scene, oculomotor, timing, and point of vantage) may be useful for the eye-tracking field and for vision scientists venturing from the lab into the wild.

## Supplementary Material

Supplement 1

Supplement 2

## Footnotes

1 We use this term for naturalistic task environments—that is, tasks that participants would perform in their natural ecological context outside the lab (regardless of whether the measurement takes place in a dedicated facility or out in the real world), with the following characteristics: (a) complex sequential behavior producing autocorrelated (and cross-correlated, potentially multicollinear) time series data; (b) unrestrained physical, or virtual, locomotion; (c) unrestrained eye–head movement (meaning the subject is actively sampling the visual world rather than being “exposed” to stimuli); and (d) occurring in a richly structured visual environment.

2 By visual field, we mean a field of view fixed to a reference frame that could be the eye (retinotopic visual field), head or head cam (craniotopic visual field), or the body/vehicle axis or velocity vector (locomotor visual field). We define it purely geometrically, not functionally, as in, for example, useful field of view (UFOV) or visual acuity/crowding tests.
