# Supplementary material for: The racer's gaze: Visual strategy in high-speed sports expertise
Source: J Vis. 2025 Jul 21;25(8):16. doi: 10.1167/jov.25.8.16 (PMC12302049; doi:10.1167/jov.25.8.16)
Supplement: Supplement 1 [file jovi-25-8-16_s001.docx]

### SUPPLEMENTARY METHODS & RESULTS

**THE RACER’S GAZE: VISUAL STRATEGY IN HIGH-SPEED SPORTS EXPERTISE**

Otto Lappi, Cognitive Science, Department of Digital Humanities, University of Helsinki

Jami Pekkanen, Cognitive Science, Department of Digital Humanities, University of Helsinki

Aleksandra Krajnc, Know-Center GmbH

Lucas Iacono, Know-Center GmbH

Adrian Remonda, Know-Center GmbH, AVL List GmbH

Eduardo Veas, Interactive Systems and Data Science, Graz University of Technology

### 1. Consistency of gaze behavior

A striking feature of both the gaze and telemetry time and distance series is the high degree of consistency: similar control action and gaze sequences are performed every time in any given bend.

A measure for degree of gaze direction consistency, absolute deviation of gaze from median gaze direction is shown in **Supplementary figure SF1.** The "median" is here calculated separately for the vertical and horizontal axis, in the display (not eye tracker) coordinate system. We will refer to this as the "central tendency position" in the visual field, for each point of vantage. (Aggregation is based on sampling by track distance (not time), i.e. based on location). In real-world eye tracking a common rule of thumb is to treat gaze points within 3 degrees of an object or location of interest as "targeting" or "fixating" said object or location. We find that the gaze median is within this distance of the central tendency position for more than 90% of track locations.


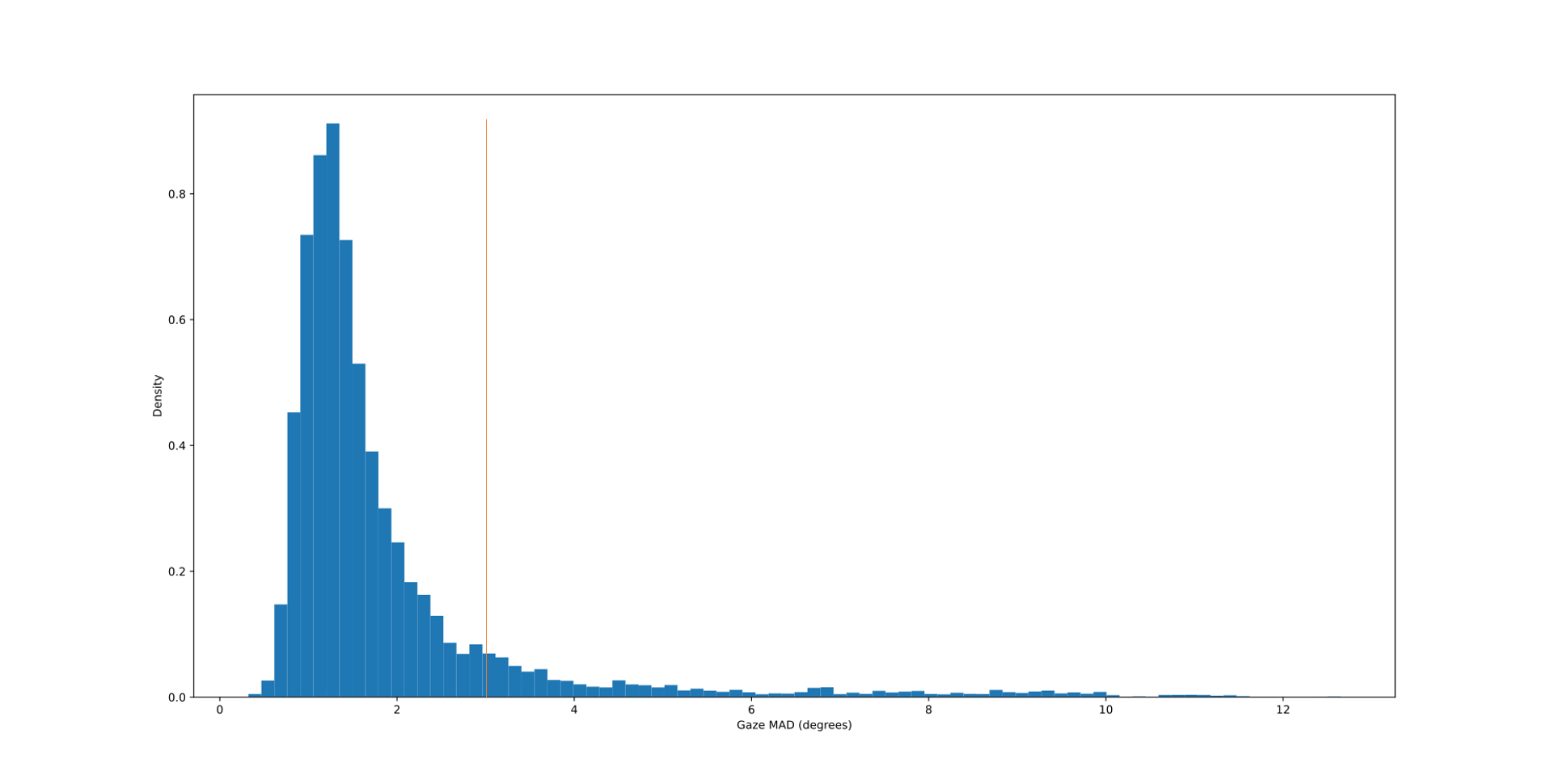


**A**


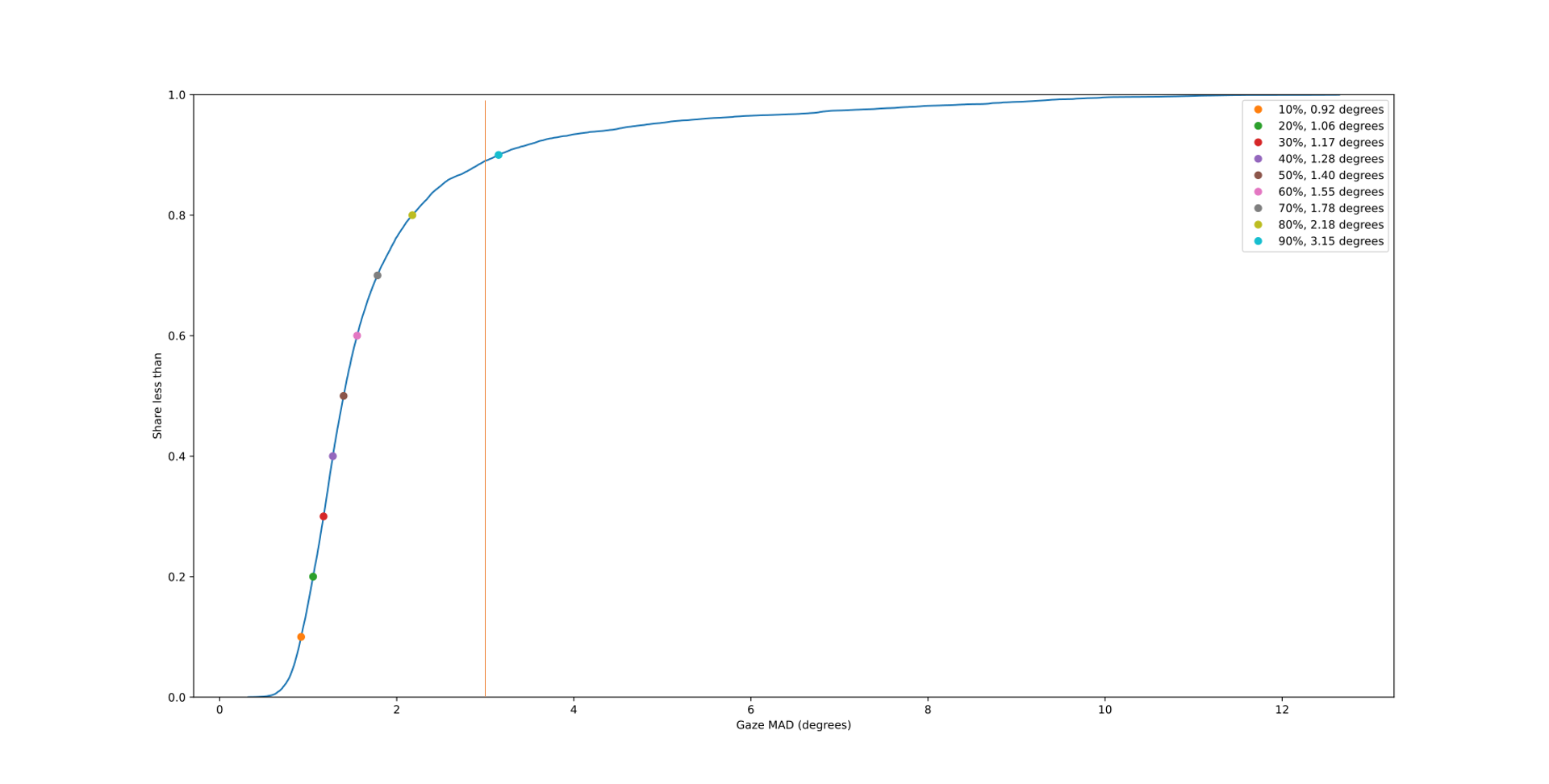

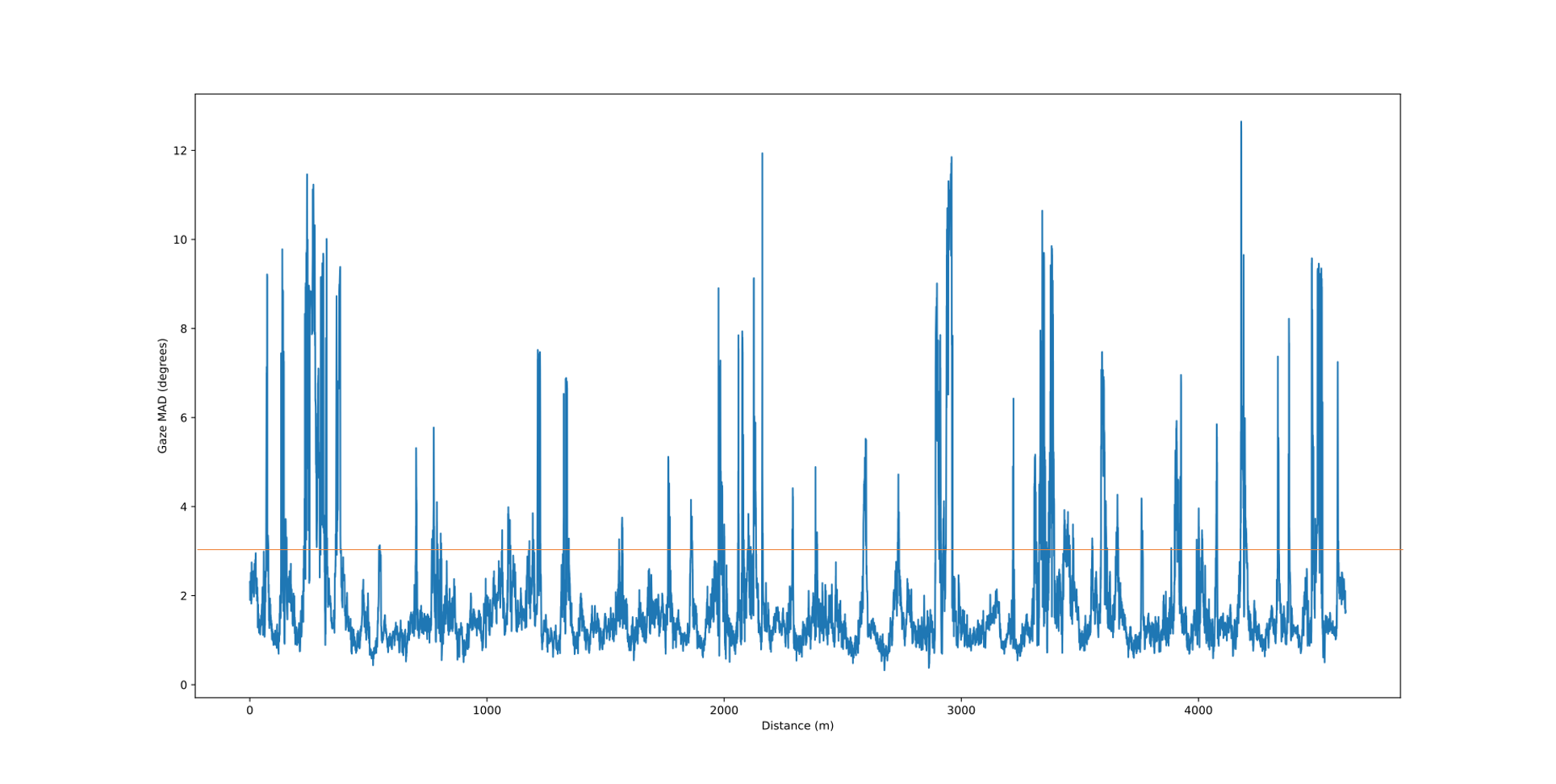


**C**

**B**

**Supplementary Figure SF 1.**

**A.** Probability density histogram of median absolute deviation. For the majority of track locations this value is within 3 degrees of the central tendency position. Although this position varies greatly between different track locations, at any given location gaze is tightly concentrated around this position.

**B.** Cumulative density function of gaze median absolute deviation from the central tendency position.

**C.** Median absolute deviation of gaze from the aggregate median position plotted against track location. Gaze is tightly concentrated especially in the bends, indicating that whatever targets the subject is fixating, he does this consistently.

### 2. Steering with the head

The oculomotor signature of the Steering with the Head strategy is the low magnitude of eye-in-head rotation relative to head and gaze rotation (i.e. head contributes most of the gaze rotation). **Supplementary table ST1** gives numerical values in our data.

SUPPLEMENTARY TABLE ST1: Deciles for absolute gaze, head and eye-in-head yaw*

| **Deciles** | **Eye-in-head** | **Head** | **Gaze** |
| --- | --- | --- | --- |
| 10 | 0.5 | 1.7 | 2.3 |
| 20 | 1 | 2.4 | 3.6 |
| 30 | 1.6 | 3.2 | 4.8 |
| 40 | 2.1 | 4.9 | 6.3 |
| 50 | 2.7 | 6.6 | 8 |
| 60 | 3.5 | 8.1 | 10 |
| 70 | 4.5 | 10.1 | 13.5 |
| 80 | 5.8 | 14.1 | 18 |
| 90 | 8 | 18.9 | 24 |
| **The yaw axis is horizontal, heading at the origin (vehicle/body/locomotor coordinate system).* | | | |

### 3. Odometer vs. Reference Path Distances

*Odometer* distance - which can be defined by path integration of location or as tyre rotation count times tyre circumference - gives the actual distance travelled on a specific trajectory. This depends on the exact line taken. Trying to determine if actions happen at specific points on the track from this signal will be accurate at the beginning of the lap (if odometer distance is re-set to zero), but localization error will accumulate over the lap distance. Error variation will be largest at the end of the run (the end of the lap if the odometer is re-set to zero at the Start-Finish line).

The cause of odometer error accumulation is lap-to-lap variation in lines taken. **Supplementary Figure SF2** shows T9 which exhibits considerable variation in the racing line taken (this can be either on purpose or due to driver error).

For simulators a noise free ground truth localization is available. **Supplementary Figure SF3** shows the ground truth *localization* of the car when the odometer reaches the same value on different laps (red dots). Even with the very high consistency of a professional driver we can see that odometer distance may drift as much as 12 m (the odometer reading at the same location can be separated by as much as 12 m). This error accumulates over the lap and the figure shows the braking zone of T12/13. A localization-based reference path (described below) offers a better method to derive the track distance metric (green dots). We used the average location of successive odometer readings, resampled to constant intervals, as the *reference path distance* measure.

The reference path was reconstructed as the average of xy (plane of travel) locations of the car This is a reasonable approximation because of the flatness of the track environment. For each lap starting from the Start-Finish line xy positions successive odometer distance observation were averaged, yielding a series of “successive average positions", i.e. the reference path. Locations on this reference path were then interpolated and resampled to equal distances between successive *reference path distance* location coordinates. The green dots in **Supplementary Figure SF3** indicate this measure is clearly the more accurate one. **Supplementary Figure SF3** shows the difference of odometer and reference path distance, indicating where and by how much error is accumulating.

Note that because the Barcelona circuit is relatively flat, we can consider the track surface points as co-planar and approximate the track as a surface embedded in a single xy plane of travel. Tracks with more pronounced elevation changes (such as ovals, or rally stages with yumps) may require considering the z axis as well.

**Supplementary Figure SF2**. Odometer distance to a given track location accumulates error due to slight differences in the exact line taken in the bends. Here T9 of Barcelona.


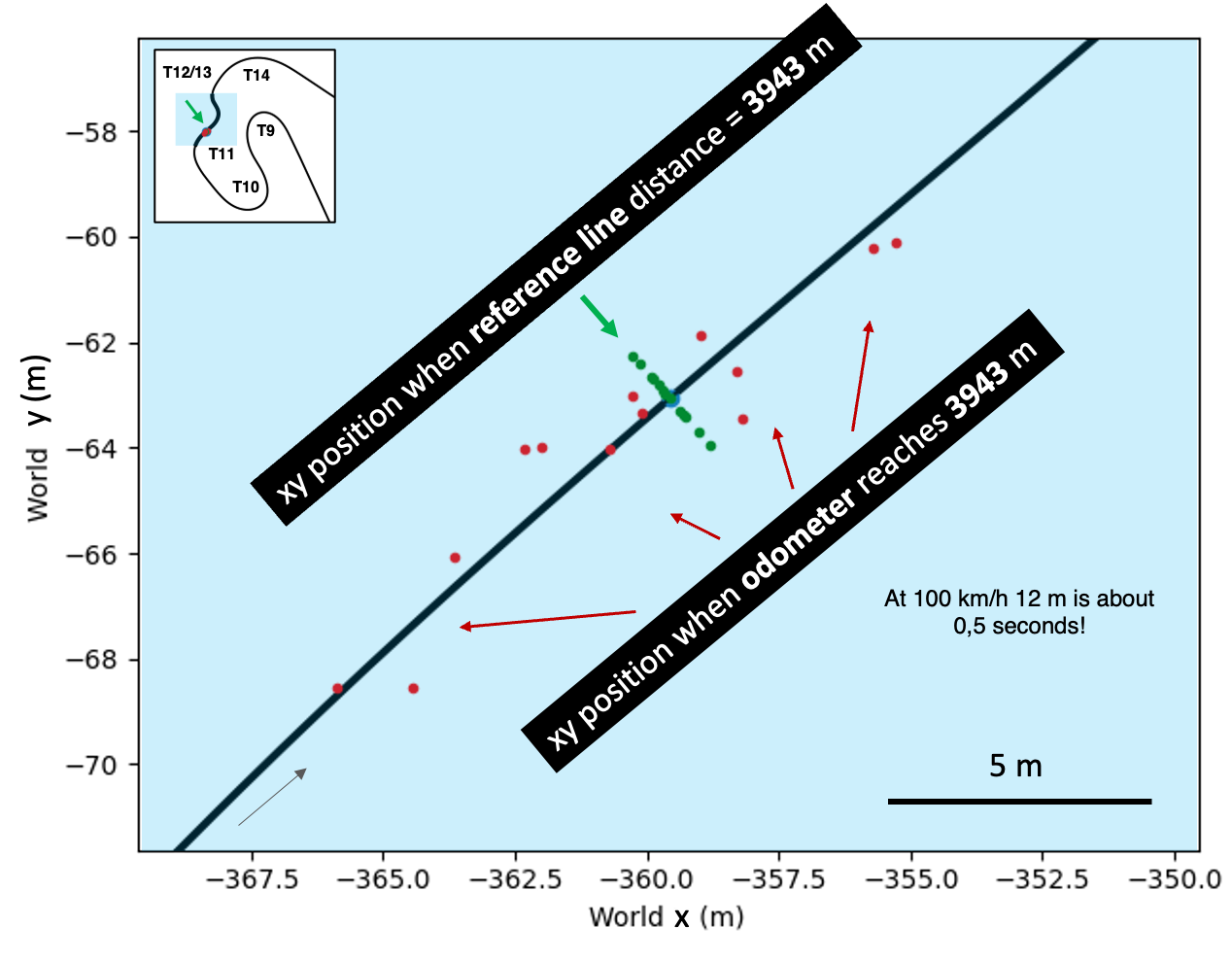


**Supplementary Methods Figure SF3.**

The magnitude of the accumulated error can amount to several meters by the end of the lap, even with a highly consistent professional driver. To avoid such drift in projecting car positions from the xy World coordinate system onto a 1-dimensional track position, we used a Track Distance coordinate system based on a reference line. Note that e.g. for evaluating localization of braking points a distance of 12m is significant. Here T12/13, the final chicane at Barcelona.


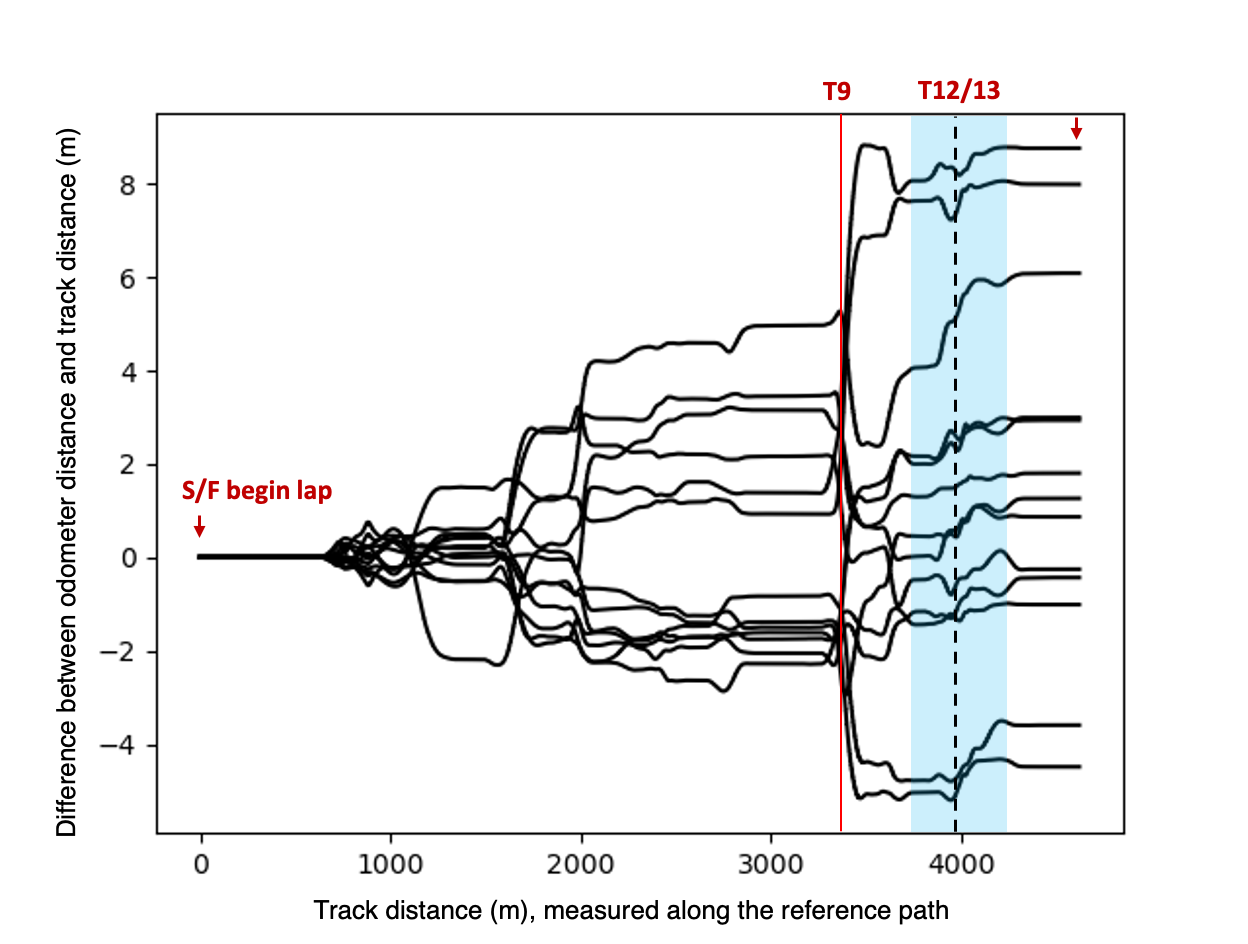


**Supplementary Figure SF4**. Accumulation of path error in odometer distance readings over a 4.7 km track (Barcelona). Each black curve represents difference between odometer value from the reference path value at successive reference path locations. T9 is a major source of odometer distance variation, that is carried over to the rest of the lap. Shaded blue area indicates the track locations in SF3.

We note that **Supplementary Figure SF2** also illustrates variation in “lane position”, relative to the reference path (not the track edges, which are not shown). We did not investigate “lane keeping” behavior i.e. lateral position, but if we had, this would be more appropriately evaluated relative to the reference path, rather than relative to the track edges or the centreline which are sensitive to changes in track width, and parts of the geometry of the track edges that would not make a difference to steering. Recovering track edge information would allow us to calculate “lateral position” relative to centreline, as is commonly done. However, for any lateral deviation the reference path is the relevant reference, as a racing line is not generally constrained by the track centre, but only one track-edge at a time. E.g. in entering a left-hand bend the racing line is mostly constrained by the left track edge – the boundary of the curve apex – and not by how much track width there is available on the right side. A speed-optimized reference line could also have been used, and this also would be a better reference than track edges, but we opted for an empirical rather than an *a priori* approach from the data we had.

### 4. Head Pose Estimation

We describe gaze direction and its decomposition into head and eye direction in an approximate vehicle longitudinal axis / body axis / locomotor frame of reference, which we call *heading* (and which is approximately the same as that of the simulator rig longitudinal axis pointing to the centre of the display screen. The vertical axis is defined as perpendicular to the yaw axis.

To estimate head pose relative to this frame of reference we used manual annotation of reference feature locations (tops of the left and right wheel rim**.** They were chosen as they are salient visual references that are clearly visible at all times. (The visual horizon e.g. is not always visible, but occluded by trackside objects). The mid-point on the reference axis is used as approximation of vehicle heading. If eye heights (real and virtual) are at the same height from the ground plane as the reference, then the "horizon" of this yaw axis will be parallel to the plane of travel. This is not exactly true, nor is the virtual camera located exactly at the car coordinate origin, or the eye tracker head cam (or the driver’s eye) exactly at the virtual camera location. We confine ourselves to analyses where these translations can be considered negligible.

Head yaw and roll and the eye-in-head contribution to gaze yaw are defined relative to this frame of reference. This axis will not be affected by chassis roll and head roll.

Note also what we are calling the “eye-in-head” yaw is therefore not the same as horizontal eye-in-head coordinate in the head mounted eye tracker’s native coordinate system. When decomposing gaze yaw into head yaw and eye-in-head yaw, the latter is *not* the horizontal coordinate in the eye tracker’s native coordinate system, but rather parallel to the yaw axis. These are not the same when the head is in roll. For the present purposes we define *eye yaw* as the *gaze yaw – head yaw* difference in the locomotor (display) coordinate system. This approximation does not exactly match the native eye-in-head values of the eye tracker (due to foreshortening from head roll, and the image rectification approximation), but simplifies analysis and interpretation.

This point may become relevant when considering the present findings in relation to more standard laboratory work on eye movement behavior.

Error sources to this coordinate transformation include suspension movement and camera fisheye distortion. From **Supplementary Movie 1** it can be seen that especially when the head is in extreme angles and the wheels turned - that is: in the bends - the longitudinal reference axis rotates by a few degrees in the direction of the bend. Head yaw and gaze yaw values relative to this reference axis should therefore be considered underestimates of the “real” yaw values relative to the chassis longitudinal axis.

Note that that the vehicle longitudinal axis (“*heading*” as the automotive and aerospace engineers use the term) only corresponds to direction of travel (“*heading*” as experimental psychologists usually use the term) on the straights. In bends the vehicle can adopt several degrees of yaw slip angle, where the chassis does not point in the instantaneous direction of velocity. For locomotor control it is the instantaneous direction of travel that would arguably provide the more “real” reference axis. We did not consider vehicle yaw slip in the analyses, so that the values are not only biased, but not strictly *heading*-referenced. This may become relevant when considering the present findings in relation to more standard laboratory work on the visual control of steering.

### 5. The Racing Line

We define Control Keypoint events/locations under the concept of the “racing line”, applyingthr concepts from vehicle dynamics engineering and elementary of driving technique (see e.g. Lopez, 2015; Milliken & Milliken, 1995; Segers, 2014). This confers some external face validity to our use of Control keypoints as a task analysis. Note that we use here only *steering wheel, brake* and *throttle* events, which are sufficient to illustrate the general concept. Other signals such as speed, lateral / longitudinal acceleration, yaw rate and gear could be used for a finer-grained performance analysis. (Cf. e.g. Lopez et al., 1997, pp. 19-55; from the point of view of vehicle telemetry signal analysis see especially Segers, 2014, pp. 143ff).

As a first approximation, the way a racing driver takes a corner can be analyzed into a small number of discrete control events which follow one another in the same order, and have standard definitions in vehicle dynamics and high-performance driving technique.

The first phase is the *Approach,* where the driver arrives to the corner at the very outer edge line of the track, at full throttle. The approach phase ends when the driver very quickly shuts the throttle and applies full brakes.

**Control action 1: Throttle lift.**

**Control action 2: Maximum braking.**

The braking phase is followed by the *Entry* phase, what begins when the steering wheel is turned:

**Control action 3: Turning in.**

During turn entry, progressively more steering wheel angle is applied and at the same time brake pedal pressure is released (referred to as “trail braking”). Fast turns may not require a braking phase, as they can be taken with just a “lift” of the throttle. Shifting down may be done during the braking and/or entry phases. As the trajectory of the vehicle cuts to the inside edge of the track, clipping the apex, the driver completely releases the brakes and gets back on the throttle.

**Control action 4: Throttle on.**

**Control action 5: Clipping the apex.**

The clipping point is a location where the car is closest to the inside region of the bend, the apex, and furthest from the centreline (On some corners the driver may “cut” beyond the inside track edge, e.g. riding the inside kerbs). As throttle is progressively applied, the steering wheel angle is reduced. In this *Cornering* phase the vehicle reaches minimum speed, maximum yaw rate and maximum lateral acceleration. We consider the cornering phase to end and the *Exit* phase to begin when the driver reaches full throttle again (and does not have to lift again e.g. due to running out of track on the exit).

**Control action 6: Full throttle.**

**Control action 7: Track-out.**

In the *Exit* phase the car is allowed to unwind, with progressively increasing path radius. (Depending on the curve geometry you may be “steering with the throttle” to simultaneously control both understeer/oversteer balance and lateral position). Finally, the racing line “tracks out” at the outer edge line of the track.

This breakdown is valid for geometrically "simple" bends. Tracks with more complex surface geometry, especially in the z direction, and detailed analysis of connected bends (where there is substantial carry-over in vehicle dynamic state and driver technique between one bend and the next) would require further elaboration of the scheme - the basic “out-in-out” cornering sequence or racing line.

**Supplementary Table ST2** describes how we operationalized this sequence in the present data.

**SUPPLEMENTARY TABLE ST2**

| **1. Throttle Lift**  The driver gets off throttle. We consider that the this begins the braking phase / zone in the control action epoching events / keypoint sequences.  *We used 90% throttle as the threshold for detecting the throttle lift epoching event.* |
| --- |
| **2. Brake**  The driver applies pressure on the brake pedal. The *Braking* phase / zone begins.  *We use 50% as the threshold value to detect the “on-brakes” event). Other types of vehicles may desire other types of braking manoeuvers*  **Notes**: With a skilled driver the brake pressure trace has a characteristic step-ramp shape. The shift from throttle lift to the initiation of braking is very fast and for a modern high-downforce racecar the step-change brake pressure to its maximum value very steep. Brake pressure is then released in a ramp-like fashion while turning in. (In our data the driver reaches a maximum of about 50-60% nominal brake value). The car is slowed down to the desired turn-in speed, and weight shift rear-to front is modulated by the brake pressure as well, giving front tyres more grip for turn-in (“trail braling”). If the turn needs to be taken at a lower gear than the preceding straight, then gears are usually shifted down during the braking phase. |
| **3. Turn-in**  The driver turns the steering wheel to start rotating the car into the bend. This begins the *Entry* phase / zone. Gears are shifted down to whatever ratio is appropriate for the speed the bend can be taken in.  *We elected not to investigate turn-in behavior at this time and did not identify a turn-in algorithmically*.  **Note**: Turn-in begins as the brake pressure is released. Due to the more gradual “rolling in” of steering a turn-in *point* cannot be identified as clearly. Turning in is a smoother, more gradual even than lift-off and braking. Turning in ends the braking phase, and begins the entry phase, yet brakes may not be completely released at this point; brakes are released as the steering wheel is turned. This technique is known as “trail braking”. The car cannot take steering lock under maximal braking – braking and turning forces must be balanced against one another, remaining within the maximum overall grip that each tyre can generate (sum of lateral and longitudinal grip is approximately constant, yielding the so-called friction circle). |
| **4. Throttle On**  The driver begins to re-apply throttle. One may consider clipping the apex or getting on the throttle as the transition from *Entry* to *Cornering*, whichever comes first in the sequence.  *We use 20% throttle to identify the throttle-on event.*  **Note**: This action is done to stabilize the car by shifting tyre loads more evenly front-to rear for maximal cornering capability and/or to give the rear tyres more “traction”, prepare for accelerating out of the bend onto the next straight. This can happen before or after clipping the apex, depending on car, trach geometry and driver preference. As progressively more throttle is applied the steering angle is unwound. |
| **5. Clipping Point**  The car is closest to the inside track edge, farthest of the centreline. The driver “cuts the corner”, taking the car to the inside edge, the car may even run over the kerb on the inside.  *We elected not to localize apex-clipping at this time*.  **Note**: We use the term Apex for the entire scene region on the inside of the bend, and the term clipping point for the location on the racing line where the inside wheels “cut” furthest into the apex. Also, the term clipping *point* – which we borrow from the domain expert jargon - may be slightly misleading as the car may spend some time “hugging” the apex (especially in a long constant radius corner like T4). Determining the apex-clipping location cannot be done on the telemetry alone, but requires use of localization data. |
| **6. Full Throttle**  The driver goes to maximum throttle opening. We consider this to begin the *Exit* phase / zone. Gears are usually shifted up.  *We used 90% throttle as the threshold for detecting the lift event.* |
| **7. Track-out**  On exiting the bend the car is allowed to drift to the very outer edge of the track. The cornering sequence is considered to end here, and the car has entered the following straight.  **Note**: In the case of connected bends that are not separated by a straight this sequence, appropriate for a geometrically simple corner, would naturally need to be extended and modified. |

Individual turns, when not connected by a long enough straight, can exhibit carry-over in performance. E.g. when the exit speed from one turn directly influences the entry speed of the next turn. Although we did not do any performance analyses, we did segment the track into “mini-sectors” for data wrangling and communication purposes. **Supplementary Table ST3** gives the start and end points of each sector in track distance coordinates. The aim was to partition the track into segments that would have minimum possible carry-over from one segment to the next.

**Supplementary Table ST3**

| Sector | Label | Begin dist | End dist | Length |
| --- | --- | --- | --- | --- |
| S1 | S/F (start) | 0 | 440 | 440 |
| S2 | T1/2/3 | 440 | 1297 | 857 |
| S3 | T4 | 1297 | 1822 | 525 |
| S4 | T5 | 1822 | 2180 | 358 |
| S5 | T6/7 | 2180 | 2550 | 370 |
| S6 | T8 | 2550 | 2875 | 325 |
| S7 | straight | 2875 | 3130 | 255 |
| S8 | T9/10 | 3130 | 3732 | 602 |
| S9 | T11, R12/13 | 3732 | 4051 | 319 |
| S10 | T14 | 4051 | 4320 | 269 |
| S11 | S/F (end) | 4320 | 4620 | 300 |

### 6. Gaze and control keypoints

**Supplementary Figure SF5** shows all the gaze and control action keypoints, separated into individual laps. Time and distance coordinates of all events are in the repository.

**
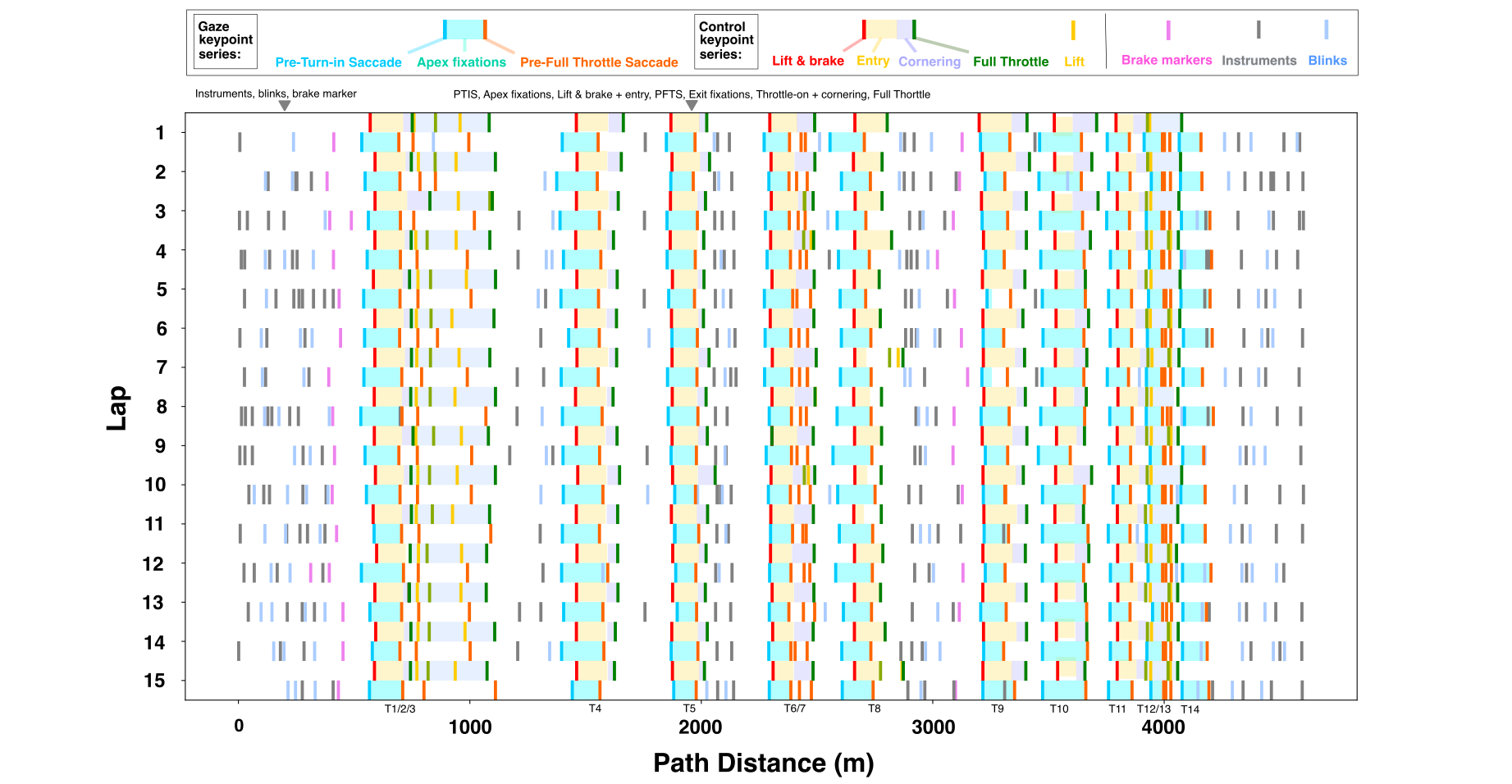
**

**Supplementary Figure SF5.**

Raster plot of lap-by-lap gaze event data and control action (telemetry) event data. The sequential organization of the racing line is established by epoching events (lift, brake, full throttle) which have a precise location on the track. *Cyan shading* indicates apex fixations, initiated by the PTIS and terminated by the PFTS. The zones where full throttle is achieved on exiting bends in shaded green.

Gaze keypoints. *Cyan*: Pre-turn-in Saccade. *Orange*: Pre-Full-Throttle Saccade.

Telemetry keypoints. *Red*: throttle lift & brake. *Yellow*: throttle lift. *Green*: full throttle. *Yellow* *shading*: entry (turn-in).

Instrument fixation (*gray*) and blinks (*blue*) are confined on the straights, where visual guidance is not critical, but are less constrained and do not occur at a specific, recurring keypoint.


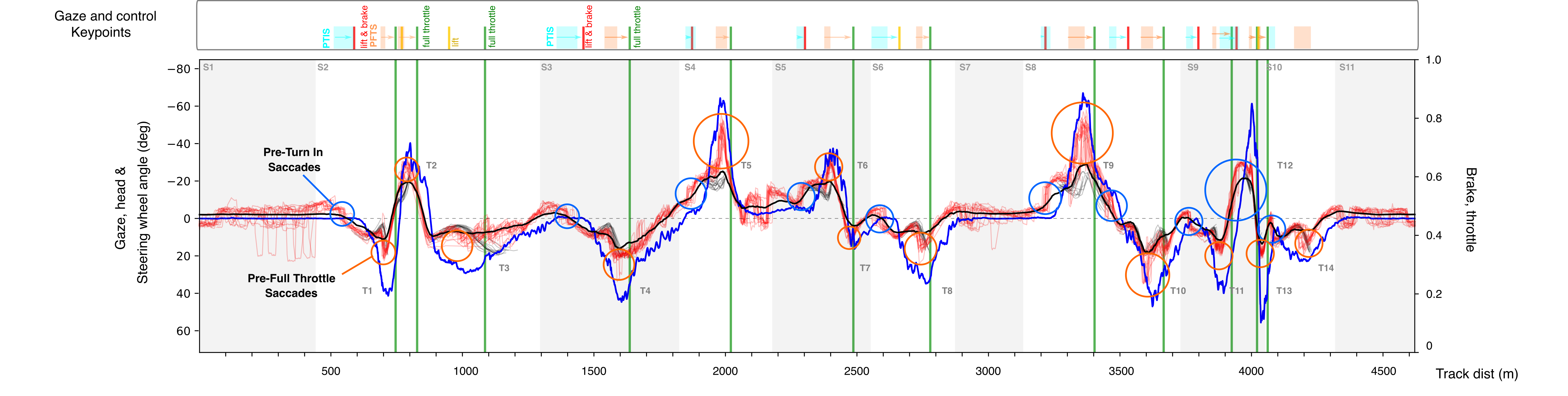


**Supplementary Figure SF6.**

Gaze, head and steering signal with PTIS and PFTS indicated. PTIS often cross the midline (shift from head-leading to eye-leading), and the PFTS take gaze to a higher eccentricity than head direction (eye-leading).


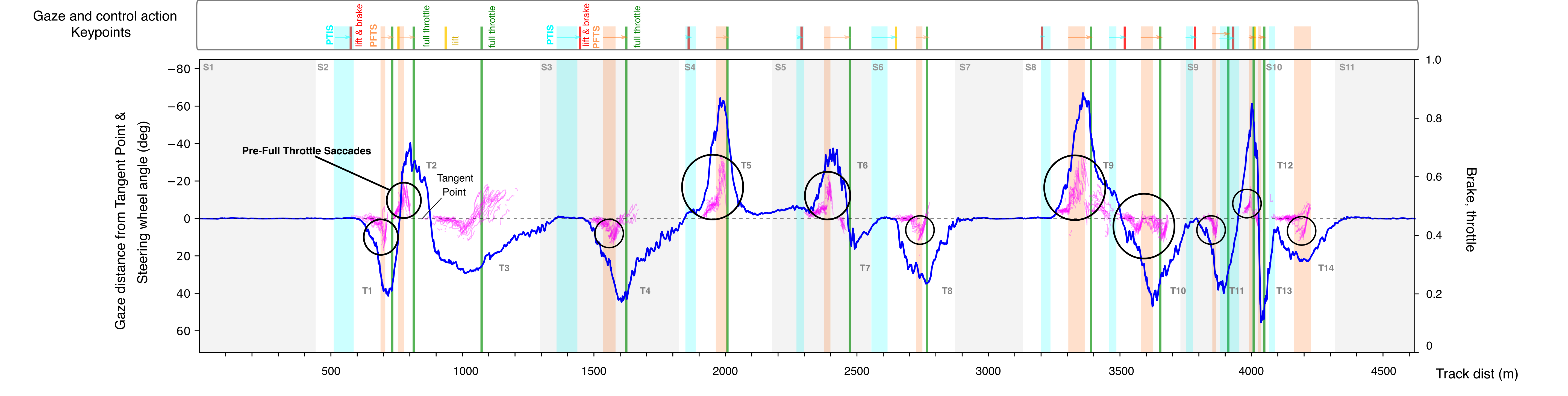


**Supplementary Figure SF7.**

Gaze, distance from the tangent point during Pre-Full Throttle Saccades. Gaze leads head, and the saccades initiating exit fixations are directed away from the tangent point..

**REFERENCES**

Lopez, C. (1997). *Going Faster! Mastering the Art of Race Driving: The Skip Barber Racing School*. Bentley Publishers.

Remonda, A., Veas, E., Luzhnica, G. (2021). Comparing Driving Behaviour of Humans and Autonomous Driving in a Professional Racing Simulator. *PLoS ONE* 16(2): e0245320 doi: 10.1371/journal.pone.0245320

Segers, J. (2014). *Analysis Techniques for Racecar Data Acquisition*. 2nd, edition. SAE International.. Warrendale, PA.
